# Established patterns of animal study design undermine translation of disease-modifying therapies for Parkinson’s disease

**DOI:** 10.1371/journal.pone.0171790

**Published:** 2017-02-09

**Authors:** Caroline J. Zeiss, Heather G. Allore, Amanda P. Beck

**Affiliations:** 1 Section of Comparative Medicine, Yale University School of Medicine, New Haven, Connecticut, United States of America; 2 Department of Internal Medicine, Yale University School of Medicine, New Haven, Connecticut, United States of America; 3 Department of Biostatistics, Yale School of Public Health, New Haven, Connecticut, United States of America; 4 Department of Pathology, Albert Einstein College of Medicine, Bronx, New York, United States of America; Stanford University School of Medicine, UNITED STATES

## Abstract

Translation of disease-modifying therapies in neurodegenerative disease has been disappointing. Parkinson’s disease (PD) was used to compare patterns of preclinical study design for symptomatic and potentially disease-modifying interventions. We examined the relationship of model, intervention type and timing, outcomes and outcome measures in 543 animal and human studies (1973–2015) across a contemporary cohort of animal and human interventional studies (n = 445), animal studies for approved interventions (n = 28), animal and human studies for those that failed to translate (n = 70). Detailed study design data were collected for 216 studies in non-human primate (NHP) and rodent toxin-induced models. Species-specific patterns of study design prevailed regardless of whether interventions were symptomatic or potentially disease-modifying. In humans and NHPs, interventions were typically given to both sexes well after the PD phenotype was established, and clinical outcome measures were collected at single (symptomatic) or multiple (disease-modifying) time-points. In rodents, interventions often preceded induction of the model, acute toxic protocols were common, usually given to young males, clinical outcome measures were used less commonly, and outcomes were less commonly assessed at multiple time points. These patterns were more prevalent in mice than rats. In contrast, study design factors such as randomization and blinding did not differ appreciably across symptomatic and disease-modifying intervention categories. The translational gap for potentially disease-modifying interventions in PD in part results from study designs, particularly in mice, that fail to model the progressive nature and relatively late intervention characteristic of PD, or that anchor mechanistic and neuropathologic data to longitudinal clinical outcomes. Even if measures to improve reproducibility are broadly adopted, perpetuation of these norms will continue to impede effective translation.

## Introduction

A commonly cited contribution to translational failure [1] is flawed design, reporting and reproducibility of preclinical studies[2,3]. Recognition of these issues has spurred dissemination of guidelines [4] that if broadly implemented, are likely to improve reproducibility of animal studies. Whether this will result in improved translation of preclinical interventional successes to the clinic is an open question. The predominant reason for failed Phase II and III clinical trials is failure to demonstrate treatment efficacy [5] A natural next question is whether demonstration of efficacy in animals is sufficiently robust prior to initiation of human trials [6,7]. If these trials are accompanied by significant potential risk to participants, the requirement to demonstrate convincing preclinical efficacy with high translational potential is of critical importance. [7,8]

A large translational gap between promising animal studies and effective disease-modifying therapies is evident for neurodegenerative diseases [9]. Because symptomatic and potentially disease-modifying therapies have distinctly different goals, it follows that their attendant study designs must be similarly distinct. Specifically, to convincingly demonstrate neuroprotection of potential value to human patients, the intervention must demonstrate a reduced trajectory of severity of the established disease over time. Parkinson’s disease (PD), specifically its motor phenotype, was chosen to explore this question. Some of the most striking examples of effective cross-species translation for symptomatic therapies have occurred in the PD field. [10–12] These achievements were critically supported by the contribution of animal studies to elucidation of basal ganglia circuitry. [13,14] However, as in other neurodegenerative conditions, a translational gap for disease-modifying therapies in PD is apparent. [15–17] This dichotomy provides an opportunity for retrospective comparison of human and animal data for both successful and failed therapies. We compared three datasets: animal studies for interventions that were eventually approved for PD, human and animal studies for those compounds that failed to translate, and a contemporary cohort of animal and human studies for symptomatic and potentially disease-modifying interventions.

## Methods

### I. Animal use data for approved and failed interventions

#### Preclinical animal model data associated with approved compounds

Using PubMed (http://www.ncbi.nlm.nih.gov/pubmed), we searched for preclinical animal data for currently approved therapies for the motor complications of PD [18,19] (S1 Table). Of 14 interventions examined, only 7 had published efficacy studies in animal models of PD prior to first reports in humans. These publications (n = 28; S2 Table) were further examined to determine the species used, details of model development, timing of the intervention, intervention mechanism of action, overall outcome, and outcome measures used to define efficacy.

#### Animal model data for compounds failing to achieve comparable efficacy in animals and humans

Next, we used the same approach to compare methodology of human clinical trial and preclinical data for 10 interventions failing to achieve comparable results across humans and animals (S3 Table). These interventions were identified from the literature [16,17,20–22].

### II. Identifying contemporary patterns of animal model use in Parkinson’s disease

#### Source data

A dataset comprised of 445 interventional studies across multiple human and animal species was aggregated from PubMed using search terms and time limits described in S4 Table.

#### Dataset generation

The following information was collected for each study: species, strain, model (for animal studies), intervention, mechanism of action (MOA, of the intervention), outcome measures, outcome and approval status (definitions in S1 Appendix). Data was collected by searching the abstract, methods or references in each paper (S1 Dataset).

- Species and animal model: Animal models were broadly categorized by toxin (e.g. 1-methyl-4-phenyl-1,2,3,6 tetrahydropyridine;MPTP or 6-hydroxydopamine; 6-OHDA, or rotenone), biological agents (lipopolysaccharide), pharmacologic models (e.g.) or genetic models.
- Intervention: Only interventional studies were included. These were defined as those in which the effect of an intervention (pharmaceutical, phytochemical, physical, genetic, behavioral or environmental) on the PD phenotype was examined. Interventions were classified as symptomatic (defined as temporary amelioration of Parkinsonian signs or complications of dopaminergic treatment without altering the course of the disease) or potentially disease-modifying based on literature reviews [16–18,23,24]. Those interventions that are already approved for PD, related interventions within a similar class, and interventions, such as exercise, that have been used in PD but so far have not been shown to appreciably alter disease course were classified as symptomatic. The remainder were classified as potentially disease-modifying (S1 Dataset).
- Mechanism of action (MOA) of the intervention: To categorize interventions by their MOA, we utilized a controlled vocabulary centered on cellular mechanisms using the Gene Ontology Project (GO:http://www.geneontology.org).[18,25] Mechanistically similar interventions were aggregated under common parent GO terms.
- Outcome measure: These were categorized as clinical or non-clinical. Clinical outcomes were defined as variables related to physical movement. In humans, outcome measures included changes in the Unified Parkinson’s Disease Rating Scale (UPDRS) [26], severity of dyskinesia and duration of “ON” and “OFF” times. In animals, clinical outcomes included a broad array of motor and behavioral tests in rodents, and scoring systems in NHPs. Non-clinical outcomes included changes in gene and protein expression, histologic, electrophysiologic, imaging or biochemical parameters.
- Outcome: Outcome was obtained from the abstract, and comprised four categories: Improved, Ineffective, Worsened, or Mixed effects (those studies with improvement in one outcome measure but worsening of another).

### III. Detailed study design analysis in 216 NHP and rodent studies

Methodologic details of studies using MPTP and 6-OHDA intoxication were collected in 92 mouse, 44 rat and 80 NHP studies (S5 Table and S2 Appendix). These toxic models were chosen as they were by far the most commonly used, and provided a consistent model against which to assess additional experimental conditions. The following data were collected as previously described by Kilkenny et al [2]: whether an ethical statement of animal use was reported, reported sex, age and strain, management (recorded as yes if three of the following four variables–diet, water, temperature and dark-light cycle—were reported), animal numbers reported in methods or results, group size, whether a sample size justification was provided, whether randomization was used to select study groups, whether observers of outcomes were blinded as to treatment status, and whether statistical tests were reported. Results were reviewed by a biostatistician. Additionally, we collected data on 6-OHDA and MPTP intoxication protocols (dose, route of administration and frequency of administration), timing of the intervention with respect to administration of the toxin, and whether outcomes were examined once, or more than once after application of the intervention.

#### Analysis

Datasets were used to compare relationships between species, model choice and details of use, intervention type and timing, outcome and outcome measure choice and timing. Results from all three datasets were reported as proportions. Studies were defined as individual published studies i.e. a single PMID. Individual studies could contain more than one instance of each variable.

## Results

### I. Preclinical animal model data associated with approved compounds

Of 14 approved interventions examined (S1 Table), animal studies were published prior to human studies in 7 (S2 Table). Twenty-eight publications (describing 34 animal studies) spanning the period from 1973–2002 were included.

#### Species and model

The two most recently approved drugs, istradefylline and rotigotine, had the largest number of studies, reported across four species each. Of the remaining four drugs, all had preclinical studies reported in rodents. The 6-OHDA model was used in 8/15 rat studies. The MPTP model prevailed in mice (4/7 studies) and non-human primates (12/12 studies).

#### Timing of the intervention

In all NHP studies, the Parkinsonian phenotype was established before the intervention was given. In rat 6-OHDA studies, the intervention was given two to three weeks after 6-OHDA administration in all but one study. Murine MPTP studies all utilized an acute or subacute protocol of administration with the intervention applied either prior to MPTP administration or within 60 min of the first MPTP dose.

#### Outcome and outcome measures

All preclinical animal studies showed improved outcomes. Clinical outcomes were reported in all NHP studies, in the majority of rat studies (13/15) and in 4/7 mouse studies. Of four studies in which neuroprotection was reported, all were reported in rodents, all relied on non-clinical measures only, and in all, the intervention was given prior to (3/4), or 60 min after toxin administration (1/4).

#### Mechanism of action of the intervention

The interventions in S2 Table constitute symptomatic therapies for PD [18]. The animal models upon which these interventions were tested exhibit motor phenotypes resulting from dysfunctional striatal neurotransmission. In the majority of studies, alteration of this same clinical phenotype was used to assess response to an intervention targeting some aspect of striatal neurotransmission. Under a third of studies (9/30) additionally reported a pharmacologic measure supporting target engagement by the intervention.

### II. Animal model data for interventions failing to achieve comparable efficacy in animals and humans

Seventy human and animal publications for 10 interventions spanning the period from 1998–2015 were included (S3 Table)

#### Species and model

Use of rodent models (15 mouse studies; 24 rat studies) prevailed over studies using NHPs (12 studies). MPTP was used in all mouse and NHP studies. In rats, the 6-OHDA model prevailed. One- and five-day MPTP protocols dominated in mice (14/15 studies), whereas more protracted MPTP protocols were used in NHPs (9/11 studies).

#### Timing of the intervention

Human trials were evenly split between patients with early and mid-stage to advanced PD. In all marmoset studies, timing of the intervention followed MPTP treatment by 6–8 weeks. In contrast, in macaques and rats, the intervention was given at various times prior to or after MPTP administration. In mice, the intervention preceded MPTP intoxication or was given simultaneously with MPTP in all studies. In two murine studies that employed chronic protocols, the intervention preceded MPTP treatment by 1–4 weeks (19476553; 17973981). In rats, the intervention was given prior to, or shortly after model induction in 15/24 studies.

#### Outcome measures and outcome

Efficacy in human and NHP studies was based on clinical outcome measures. In rats, clinical measures were reported more commonly (18/24 studies); however, some of these were indicators of memory and cognition rather than motor function. In mice, non-clinical outcomes only were used to assess efficacy in the majority (11/15) of studies.

Interventional success was relatively poor in humans (no effect in 12/20 studies), relatively good in macaques (8/11 studies demonstrated improvement or prevention of clinical signs), and very good in marmosets and rodents (3/3 studies in marmosets, 11/11 studies in mice, and 17/18 studies in rats demonstrated improved outcomes). In macaques, outcomes were most promising if the intervention preceded or shortly followed MPTP treatment (3/11 studies), or was given simultaneous with L-DOPA treatment (4/11 studies).

#### Mechanism of action of intervention

The majority of the compounds listed in S3 Table were potential disease-altering neuroprotective agents, however two symptomatic interventions, pardoprunox and preladenant, allowed comparison to approved drugs of similar class. Of these, pardoprunex was efficacious in marmosets (three studies), rats and humans, but induced adverse effects in humans indicating that animal data predicted efficacy, but not toxicity of the compound. Preladenant achieved conflicting outcomes across three trials in humans [27], and in all of the successful preclinical studies, the drug was given coincident with model induction, (20655910) or together with L-DOPA (19332567; 20655910). AAV2-neurturin demonstrated consistently promising efficacy across rodent and NHP models—failure to replicate this in human patients has been ascribed to challenges of product delivery,[28] relatively greater nigrostriatal and axonal compromise in human PD patients compared to MPTP and 6-OHDA induced animal models, [21,22] and failure to address the degenerative drive in PD induced by alpha-synucleopathy. [22,29]

Several conclusions can be made from comparison of these two datasets (S2 Table: approved interventions and S3 Table: interventions that failed to translate). Their obvious distinction resides in the intended goal (symptomatic vs. disease-modifying) of their constituent interventions. In both datasets, toxin-induced (MPTP or 6-OHDA) preclinical models were equally utilized, and in both, predominantly promising outcomes were achieved. However, in failed interventions, application of the intervention preceded PD model induction more commonly, commensurate with the higher proportion of rodents used in these studies. Similarly, choice of outcome measures to assess efficacy were less heavily weighted towards clinical outcomes in these interventions. Next, to assess these patterns in contemporary studies, we used a 445-study dataset (S4 Table and S1 Dataset) to explore the relationship of species, model, the type of intervention (symptomatic or disease modifying), its timing, and choice of outcome measures.

### III. Identifying contemporary patterns of animal model use in Parkinson’s disease

A total of 445 animal and human studies (derived from 425 individual publications) were included (S1 Dataset), of which 179 examined symptomatic interventions and 266 potentially disease-modifying interventions.

#### Species and model use

**Fig 1:**
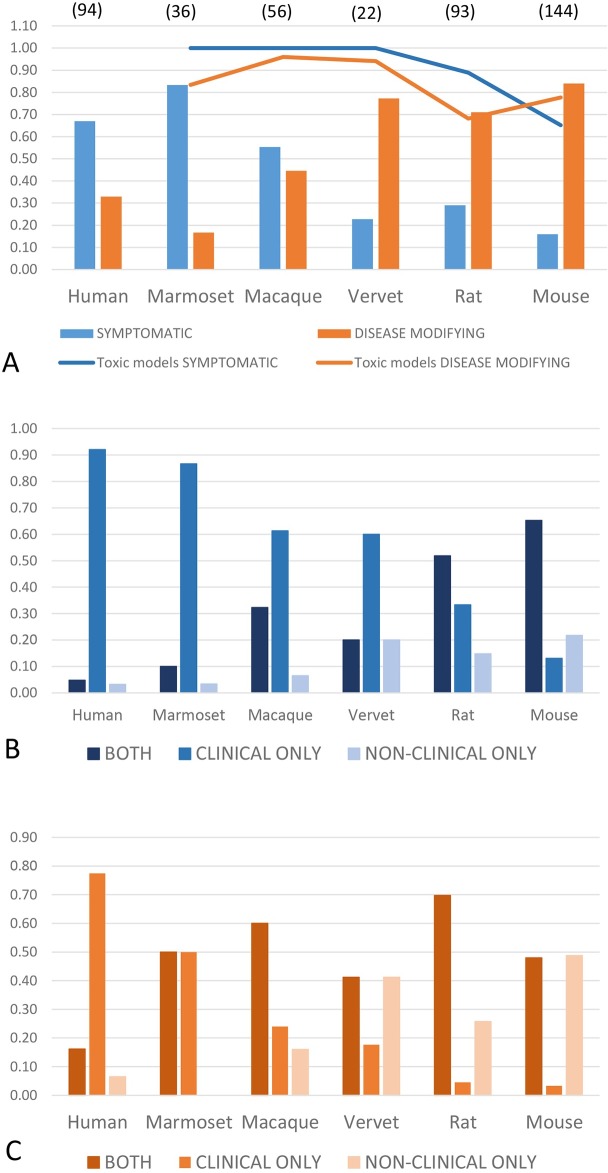
Distribution of species and model use, and type of outcome measures by intervention type. (A) Species and model use for symptomatic and potentially disease-modifying interventions. Symptomatic therapies are most commonly tested in humans, marmosets and macaques, while potentially disease-modifying interventions predominantly utilize rodents. Vervet monkey studies in this dataset were most commonly used to explore cell-based therapies. Across all animal species, toxic models (defined as those induced by MPTP or 6-OHDA, and indicated by the blue and orange lines respectively) prevailed, and were the predominant model type used regardless of intervention type. Numbers in parentheses indicate total number of unique studies by species in the dataset. (B) Type of outcome measure by species, for symptomatic therapies. The majority of human and NHP studies report a clinical outcome only (mid-blue column). The proportion of purely clinical outcomes declined progressively in NHPs, rats and mice, while the proportion of purely non-clinical outcomes (light blue column) reported increased correspondingly across the same species. Studies reporting both clinical and non-clinical measures were most common in rodents (dark blue column). Numbers of studies for each species as in 1A. (C)Type of outcome measure by species, for potentially disease-modifying therapies. While clinical outcomes only prevail in humans (mid-orange column), a higher proportion of both clinical and non-clinical outcome measures are collected in NHPs (dark orange column). This pattern also prevails in rats, however in mice, 50% of studies utilized non-clinical measures only (light orange column). Numbers of studies for each species as in 1A.

In humans, marmosets and macaques, symptomatic interventions were examined most commonly. In contrast, rodents and vervet monkeys were predominantly employed to test potentially disease-modifying therapies. Toxic models (defined as induction of a Parkinsonian phenotype by MPTP or 6-OHDA) were most commonly used across all animal species regardless of intervention type (Fig 1).

#### Outcome by intervention type and species

For symptomatic therapies, improved outcomes prevailed across all species (with the exception of vervet monkeys). For disease-modifying interventions, the lowest success rates were evident, as expected, in human studies (32% had an improved outcome), with increasing success rates in NHPs (67–80%) and rodent studies (90% and 95% improved outcomes in mice and rats, respectively; Table 1).

**Table 1:** Outcomes by species and type of intervention (n = 445 studies).

		Improved (%)	Ineffective (%)	Mixed (%)	Worsened (%)
Human	Symptomatic (n = 63)	73	21	5	0
	Disease modifying (n = 31)	32	68	0	0
Marmoset	Symptomatic (n = 30)	87	4	17	3
	Disease modifying (n = 6)	67	17	0	17
Macaque	Symptomatic (n = 31)	90	0	10	3
	Disease modifying (n = 25)	84	12	4	4
Vervet	Symptomatic (n = 5)	60	0	20	20
	Disease modifying (n = 17)	88	6	6	0
Rat	Symptomatic (n = 27)	81	0	7	7
	Disease modifying (n = 66)	95	5	0	5
Mouse	Symptomatic (n = 23)	78	0	9	13
	Disease modifying (n = 121)	90	5	0	15

Outcomes are given as a proportion of total symptomatic or disease-altering studies.

#### Use of clinical and non-clinical outcome measures by intervention type and species

Most human studies reported a clinical outcome only, regardless of intervention type. For symptomatic interventions, outcomes included clinical measures in the majority of studies across all species. Non-clinical outcome measures alone were used most commonly in rodents. This was particularly evident in mouse studies examining potentially disease-modifying therapies–over 50% of such murine studies based outcome conclusions on non-clinical measures alone (Fig 1).

#### Distribution of intervention types across species

**Fig 2:**
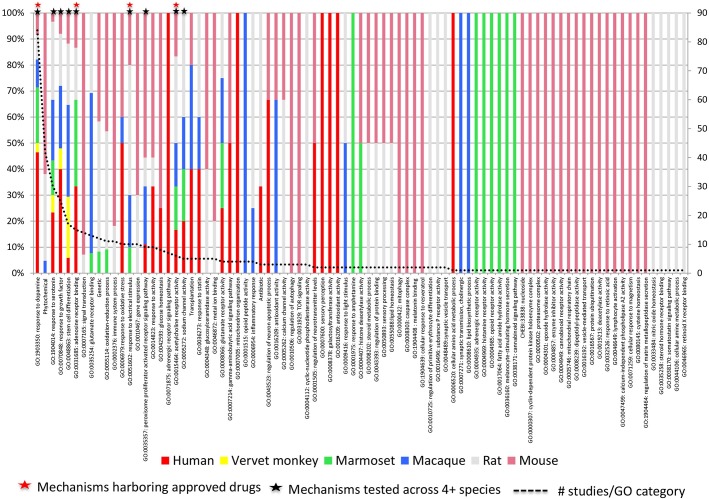
Relationship between approval status, number of studies across species and interventional mechanism of action. Interventions spanning 85 mechanisms constitute the dataset (each column represents a mechanism defined by the Gene Ontology). Column colors denote the proportion of studies done in each species i.e. greatest species diversity is indicated by the most colorful columns. A positive association is seen between studies in which related interventions (i.e. those clustering within a similar mechanism defined by a single GO term) have been tested across four or more species (9 mechanisms; black asterisks), approval status of interventions within that mechanism (red asterisks) and the total the number of studies done within that mechanism. **Red asterisks:** Mechanisms harboring interventions approved for PD. GO:1903350 response to dopamine: Apomorphine, Entacapone, Selegiline, Piribedil, Pramipexole, Rasagiline, Ropinirole, Rotigotine, Amantadine, L-DOPA and formulations, Safinamide, Benserazide. GO:0051602 response to electrical stimulus: Deep brain stimulation—various locations (STH). GO:0015464 acetylcholine receptor activity: Pro-cholinergics. GO:0031685 adenosine receptor binding: Istradefylline (KW-6002)**. Black asterisks:** Mechanisms harboring interventions tested across four or more species. GO:1903350 response to dopamine. GO:0015464 acetylcholine receptor activity. GO:1904014 response to serotonin. GO:0070848 response to growth factor. GO:0048863 stem cell differentiation. GO:0035357 peroxisome proliferator activated receptor signaling pathway. GO:0005272 sodium channel activity.

Interventions spanning 85 mechanisms constituted the dataset. The high proportion of positive outcomes for disease-modifying interventions in rodents (Table 1) implies that either an extremely wide range of mechanisms can be successfully engaged to address PD, or that study design factors may contribute to these positive outcomes. A positive association was seen between diversity of species examined within a mechanism, approval status of constituent interventions, and the total number of studies done within that mechanism. Dopaminergic mechanisms harbored the greatest number of approved interventions (Fig 2).

### IV. Study design in NHP and rodent species employed in MPTP and 6-OHDA studies

**Fig 3:**
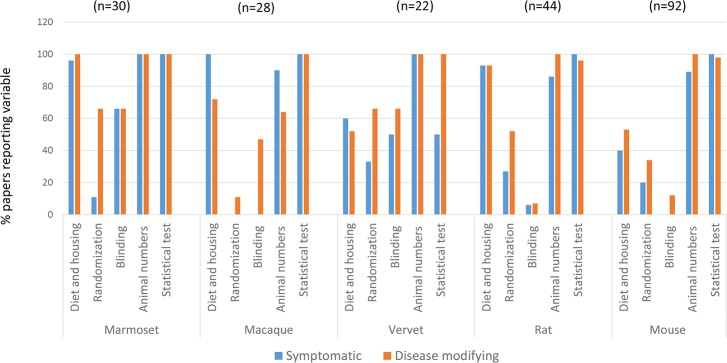
Study design factors reported across species and intervention category. Details of diet and housing were highly reported in rat and marmoset and macaque NHP studies, and least reported in symptomatic (40%) and disease modifying (53%) studies in mice. Reporting of randomization and blinding was variable across species; these data were more frequently reported in disease-modifying interventions. Reference was made to the total number of animals used, or group sizes in all NHP studies, all rodents studies for disease-modifying therapies and the majority of those testing symptomatic interventions. Statistical tests used were reported in almost all studies across species–the apparent low reporting of these tests in symptomatic studies using vervet monkeys reflects the several studies with qualitative morphologic outcomes. Numbers in parentheses indicate total number of unique studies by species (taken from S5 Table).

Because variations in study design and reporting have been implicated in poor reproducibility, [2,3,30] we collected these data in a subset of 216 MPTP and 6-OHDA studies. Details of diet and housing were highly reported in rat and NHP studies, and least reported in symptomatic (40%) and disease modifying (53%) studies in mice. These data were also relatively poorly reported in vervet monkeys. Male bias was noted for disease modifying interventions in vervet monkeys, otherwise no sex bias and a broad range of ages were seen in remaining NHP studies. There was however, a tendency towards use of younger adults (S5 Table). Murine MPTP studies were overwhelmingly done in young male mice of C57BL and related strains. A broader array of rat strains were used, but as in mice, young adult male rats predominated. These patterns tracked with species, regardless of whether interventions were symptomatic or disease modifying (S5 Table). Ethics statements regarding humane animal use were provided in 98% (disease-modifying interventions in mice) to 100% of papers (all other categories). Statistical tests used were reported in almost all studies across species (Fig 3, S5 Table). A measure of variation was provided in the majority of papers except some describing disease-modifying studies in mice and macaques (95% and 81% respectively). Reference was made to the total number of animals used, or group sizes in all NHP studies, all rodents studies for disease-modifying therapies and the majority of those testing symptomatic interventions (Fig 3). Sample size calculations or justification of animal numbers were uniformly absent. Group sizes were consistent by species, regardless of whether interventions were symptomatic or disease-altering. Group sizes ranged from ~5 to 6 in NHPs, ~8 in mice and ~8 to 10 in rats (S5 Table). Reporting of randomization and blinding was variable across species (Fig 3). As the majority of papers did not report information needed to assess whether appropriate statistical tests were used (sample size justification, distribution characteristics of the data, or Type I and II error rates), a judgment was not made on whether statistical tests were appropriate or not. Although a variety of methods to account for multiple comparisons were applied, rarely were multivariable models used or correlation of multiple outcomes on the same animal accounted for.

**Fig 4:**
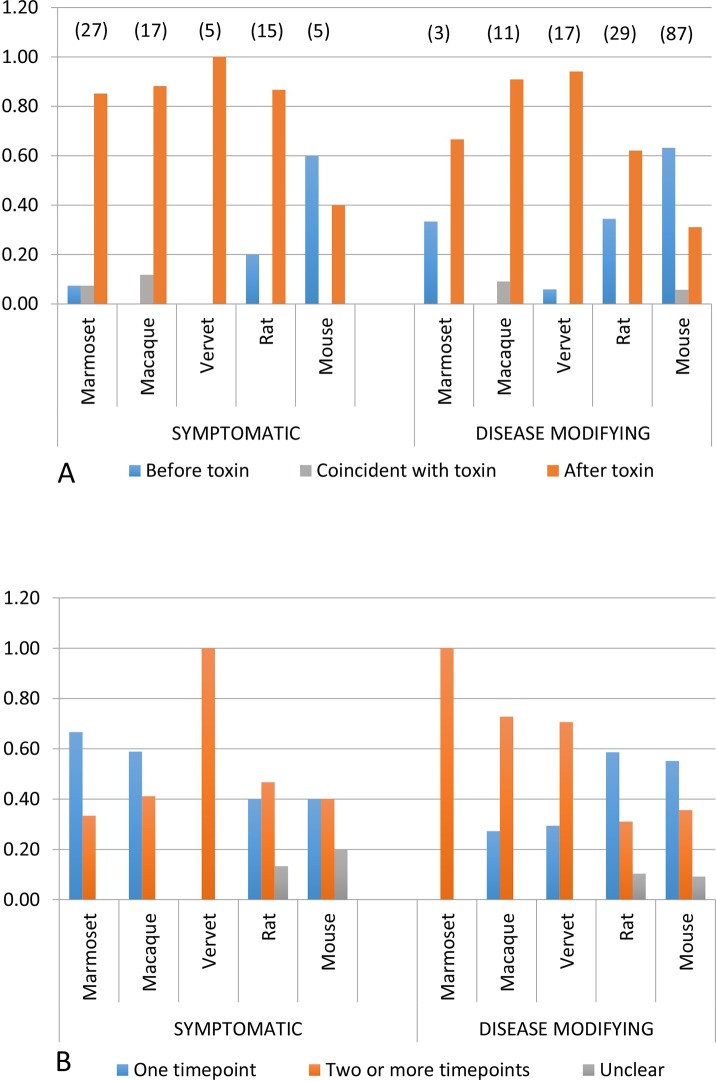
Timing of intervention with respect to toxic model induction, and number of outcome time points. (A) Timing of intervention with respect to model induction, by species and intervention type. In NHPs and a lower proportion of rats, the intervention is given after model induction regardless of intervention type (orange bar). In mice, application of the intervention precedes (blue bar) or is coincident with (grey bar) the toxin, particularly in studies for disease-modifying interventions. Numbers in parentheses indicate total number of unique studies by species (taken from S5 Table). (B) Number of outcome time points, by species and intervention type. In NHPs, outcomes are assessed more commonly at a single time point (blue bar) for symptomatic interventions, and most commonly at more than one time point (orange bar) in studies for disease-modifying therapies in NHPs. However the majority of rodent studies assess efficacy for disease-modifying interventions using only one time point (blue bar). Numbers in parentheses indicate total number of unique studies by species (taken from S5 Table).

Next, we assessed the relationship between species, category of intervention, the timing of the intervention with respect to model induction, and whether outcomes were measured once, or repeatedly (Fig 4). The distribution of species across symptomatic and disease-modifying interventions was similar to that seen in the 445 study dataset (S1 Dataset), with rodents predominantly used to test potentially disease-modifying therapies. In >80% of studies NHPs, regardless of intervention type, MPTP dose and frequency was titrated over days to weeks until Parkinsonian signs stabilized, after which the intervention was given. The majority of murine MPTP studies for disease-modifying interventions utilized acute one (38%) or five day (29%) protocols, and the intervention preceded (by an average of 7.5 days) or was given simultaneously with MPTP in 69% of studies. In rats, the intervention was given prior to or concurrent with 6-OHDA in 33% of animals. Outcomes were measured at a single time point in the majority of macaque and marmoset studies for symptomatic therapies, and at two or more time points for disease-modifying interventions across all NHP species. In rodents, outcomes of disease-modifying interventions were most commonly measured using a single time point (Fig 4).

## Discussion

Our data confirm the previously noted [15,17] translational gap for disease-modifying therapies for PD. By evaluating patterns of animal model use in over 500 studies, we were able to identify species-related patterns of study design that are likely to contribute to this gap (Table 2). These patterns include study design factors that affect the extent to which to which a causal conclusion within a study is warranted (internal validity), and the extent to which these conclusions can be generalized to other situations (external validity)[1,31].

**Table 2:** Summary of identified study design findings and proposed solutions to promote robust evidence for neuroprotection.

Finding	Proposals to promote robust evidence for neuroprotection
Factors affecting internal validity	
1. Details of diet and housing least reported in murine studies	Report details of diet, housing and husbandry [4]
2. Poor reporting of blinding or randomization (all species)	Report methods of random allocation to study groups and blinded observation of outcomes [4,32]
3. Insufficient data reported to assess appropriateness of statistical test	Report sample size justification, distribution characteristics of the data, Type I and II error rates [4,32]
4. Relatively small group sizes (~5 to 6 in NHPs, ~8 in mice and ~8 to 10 in rats)	These may be supported after appropriate sample size calculation [33,34]
**Factors affecting external validity**	
1. Murine studies predominantly performed in young male mice of C57BL/6 strain	Use both sexes, older animals, variety of strains and models
2. Heavy reliance upon acute, non-progressive toxic models (MPTP, 6-OHDA)	Utilize a variety of mechanistically diverse models, incl. those with a progressive phenotype if the intent is to demonstrate neuroprotection
3. Application of intervention prior to or concurrent with model induction by toxin, particularly in mice	Establish the phenotype before application of the intervention
4. Single outcome time-point, particularly in rodents	Utilize a longitudinal study design if the intent is to demonstrate neuroprotection
5. Tendency to utilize non-clinical outcomes to demonstrate efficacy, particularly in mice	Collect non-clinical mechanistic outcome measures together with longitudinally recorded clinical measures to associate target engagement with neuroprotective potential

### Study design and reporting in NHP and rodent toxin-induced models

Toxin–induced (6-OHDA and MPTP) models were chosen for comparison as they are the most commonly used models in preclinical PD studies, and provided a consistent model against which to assess additional experimental conditions. As noted in other studies, reporting of randomization and blinding was variable [2,33,35,36]. Reference was made to numbers of animals used in methods or in results in the majority of papers, however sample size calculations or justification for selection of group sizes for a predefined treatment difference were not provided. Group sizes were consistent by species, regardless of whether interventions were symptomatic or disease-altering. Group sizes were often fewer than 6 in NHPs, and 10 in rodents. While these sample sizes are small, and have been reported to undermine the reliability of studies [33], Janusonas [34] describes that with some methods, sufficient power can be achieved with such small sample sizes. Commonly, unadjusted parametric statistical tests were reported across species and study category; however, insufficient information was provided to make an accurate assessment of their appropriateness. Failure to account for additional factors associated with the outcome with multivariable modeling or indeed any of the current modeling methods of biologic processes may be further reasons leading to failures in translation. As shown in Fig 3, reporting of many of the study design factors was equivalent across intervention categories (symptomatic and disease-modifying). In fact, reporting of some factors was more frequent in studies for disease-modifying interventions. This suggests that disparities in those aspects of study design and reporting previously described by other authors [2] do not account for the larger translational gap noted in disease-modifying compared to symptomatic therapies in PD. This prompted us to examine additional variables that may influence face validity of models used for symptomatic and disease modifying interventions. Face validity is defined as the extent to which the model recapitulates key symptoms, neuroanatomical pathology and neurophysiological responses of the human disease [37]. Specifically, we examined the relationship between intervention type (symptomatic vs. disease modifying), methods of model induction, timing of the intervention, outcome, type of outcome measures and whether outcome measurements were collected once or more frequently.

### Translation of symptomatic therapies

The success rate for symptomatic interventions was high in humans (73%), and was preceded by high success rates in NHPs (particularly marmosets) and rodents. At the root of this success is an understanding of the consequences of dopaminergic cell loss on striatal circuitry. [13,18] Establishing the Parkinsonian phenotype before the intervention is given (the most common approach in NHPs [38]) approximates the clinical reality of PD in which patients are treated after significant dopaminergic loss has already occurred. [39] Motor signs in humans and toxin-induced animal models derive from resulting disequilibrium of indirect and direct striatal circuitry and exacerbation of these events by L-DOPA. [18,40] Because symptomatic interventions are directed at aspects of this dysregulated circuitry,[18] use of clinical outcome measures is sufficient to demonstrate a causative relationship between the intervention, its mechanism of action and amelioration of disease phenotype. Clinical outcomes prevail across all species used to test symptomatic interventions (Fig 1) but are examined at one time point in most species (Fig 2). As most models employ dopaminergic toxins that create a non-progressive phenotype [38,41], this approach is sufficient to demonstrate symptomatic efficacy. Outcome measures in humans often include changes in the Unified Parkinson’s Disease Rating Scale (UPDRS), which can be related to values that define the minimally clinically important difference for human patients [42]. In NHPs, clinical outcome measures are also defined by well-characterized motor scoring systems. [38] Therefore in humans and NHPs, both clinical effect sizes that can be related to the extent of dopaminergic cell loss, and measures of statistical significance, are used to draw conclusions regarding efficacy. In rodents, a much wider range of motor and behavioral tests [43], were used to assess efficacy. NHP studies for successfully approved drugs typically used younger adults, indicating that age was not critical to test efficacy within the limits of this model paradigm.

### Translation of disease-modifying therapies

Neuronal loss in PD is significant (30–50% of nigral neurons) and is estimated to be begin about five years prior to presentation. [39,44]. Therefore, for both symptomatic and potentially disease-modifying therapies, application of the intervention after dopaminergic cell death has been achieved is an important aspect of face validity. While this was achieved in NHPs and rats regardless of type of therapy, in mice, the intervention preceded or was given concurrently with MPTP in the majority of studies, regardless of intervention type. Additionally, the majority of mouse studies use acute 1 or 5 day protocols. In mice, neuroprotective outcomes are favored in models using an acute MPTP protocol [45] and are strongly influenced by timing of the intervention.[46] These observations suggest that improved outcomes are influenced by transient cellular events associated with acute toxicity, and preventive or early treatment. [47] To convincingly demonstrate translationally promising neuroprotection, the intervention must be shown to delay the trajectory of clinical decline over time. While two or more outcome time points were utilized in NHP studies for disease-modifying interventions, in the majority of similar rat and mouse studies, outcomes were measured at only a single time point. Further, in mice, non-clinical outcome measures were used to determine efficacy in over half of studies for disease-modifying interventions. Rodents have the advantage that tissue availability allows collection of valuable pharmacologic and mechanistic data. However, if these are not collected over time and modeled with longitudinally recorded clinical measures, these data cannot be reliably associated with target engagement or neuroprotective potential. In rodents, the majority of studies were performed in young, male animals (of one strain, C57/BL, in mice). While this is a demonstrated means of achieving reproducible results [41] it is unlikely to predictively model cellular processes in older human patients of both sexes. These approaches predominated in mice, the most commonly used experimental species, across all three datasets, despite minimal evidence for translational success (7 mouse studies preceding approval of three drugs; S2 Table).

Interventions are typically tested in rodents prior to extending these studies to higher species. This is an ethically appropriate approach and has been successfully used in developing symptomatic therapies for PD. Why has this approach been ineffective for disease-modifying interventions? We propose three reasons.

First, we identified many of the same previously identified study design issues (particularly in mice) that contribute to overly rosy reporting of efficacy in this species [30,48,49]. The second reason is the well-recognized challenge of modeling the molecular complexity and progressive nature of PD in animals [50]. Even in NHP models in which comparative neuroanatomy favors translation of surgical techniques, ongoing cellular dysfunction in PD neurons may result in impaired distribution of, and response to neurotrophic factors that achieved good responses in NHPs.[21,22]. Extending results from animals to humans is further complicated by greater variability in human populations created by because of lifetime exposures, genetic diversity and multi-morbidity. The mitochondrial complex 1 deficiency described in PD can be induced by MPTP and other toxins [51], however progressive neuronal loss in PD is driven by multiple cellular events [52–54] that can be modeled individually in rodents. [45] Rather than relying upon the prevailing acute MPTP models in mice, the complexity of human disease can be more closely approximated by combining different reductionist models in animals. These include chronic progressive nigrostriatal oxidative or toxic damage [55–57], mitochondrial DNA damage [58] and abnormalities of protein aggregation seen in alpha-synuclein transgenic or virally transfected animals [59,60]. None of these models are perfect, but taken together, a group of studies across different experimental systems and species would provide stronger evidence for translation [3,61]. This approach is reminiscent of the guidance provided by the Animal Rule [62]. In effect, it approximates the complexity of human disease by combining slightly different reductionist models. If broadly achieved across rodent models, this approach may further reduce the use of NHP models, with the exception of therapies requiring surgical intervention, and first-in class therapies.

Finally, it appears that accepted study designs traditionally used in each species have been applied regardless of whether the intervention is symptomatic or potentially disease-modifying. This is likely to result from perpetuation of previously published methods, and the possible misconception that validity is inherent in the model, rather than how it is used. This has resulted in the tendency for rodent studies to commonly fail to assess translationally key aspects of effective neuroprotection: the capacity of the intervention to delay clinical decline of a progressive degenerative process in older animals of both sexes over time. Further, to convincingly demonstrate translationally relevant neuroprotection, the intervention must produce promising results across a range of mechanistically diverse models, using study designs that model the progressive nature and relatively late intervention characteristic of PD in humans, and that anchor mechanistic and neuropathologic data to longitudinal clinical outcomes. [63–65] These patterns prevailed across all of our datasets, and imply that accepted murine designs are re-used repeatedly with minimal evidence for translational success. While this approach may be sufficient to demonstrate involvement of a given mechanism in the disease process, it is insufficiently robust to translate to more complex systems.

Rodent studies have provided an enormous contribution to our understanding of disease mechanisms, and recent guidelines to address study design flaws [2,3,66,67] that impede reproducibility are welcome. However, even with these improvements, which are likely to improve reproducibility of animal studies, translating results from reductionist approaches to more complex systems will remain a significant challenge. Rodents are used primarily to demonstrate that a mechanism has potential therapeutic value (exploratory or proof-of-concept studies), with less emphasis placed on study design aspects that are directed towards confirmation of translational potential. Design rigor that supports internal validity is a necessary foundation for all studies. Additionally we suggest that study designs that place greater emphasis on translational relevance, utilization of mechanistically diverse models, and extension across species where appropriate, are likely to improve generalizability of approaches from animals to humans. This is likely to require reexamination of accepted norms of what constitutes a “good” model in individual fields. It would be unreasonable to expect every research paper to achieve all of the goals suggested in Table 2. However, to move the field towards translation, and prior to initiating human trials for a given intervention, an unbiased and critical assessment of the evidence that, in aggregate, provides convincing support for the overall therapeutic approach does need to be established [7,68]. This would raise the bar for investigators, funding agencies, reviewers and regulatory bodies alike.

## Supporting information

S1 Appendix Abbreviations and Definitions. (DOCX)

S2 Appendix (DOCX)

S1 Dataset (XLSX)

S1 Table Studies in animal models of PD prior to first report in humans (PubMed). (DOCX)

S2 Table Methods of animal model studies for drugs approved for PD, performed prior to approval date (PubMed; n = 28*). (DOCX)

S3 Table Comparison of clinical trial and preclinical data for compounds failing to achieve comparable results across humans and animals PubMed; n = 70*). (DOCX)

S4 Table Interventional studies in human and non-human animals. (DOCX)

S5 Table Methodologic details in MPTP (mice, non-human primates) and 6-OHDA (rats) models by intervention type. (DOCX)
