# Supplementary material for: Established patterns of animal study design undermine translation of disease-modifying therapies for Parkinson’s disease
Source: PLoS One. 2017 Feb 9;12(2):e0171790. doi: 10.1371/journal.pone.0171790 (PMC5300282; doi:10.1371/journal.pone.0171790)
Supplement: S1 Appendix — (DOCX) [file pone.0171790.s001.docx]

**S1Appendix: Abbreviations and Definitions**

**Abbreviations**

1^O^M: Primary outcome measure

3NP: 3-nitropropionic acid

6-OHDA: 6-hydroxydopamine

AIMs scale: Abnormal involuntary movement scale

Bid = twice dailyD2R: Dopamine receptor D2

GO: Gene Ontology Project

LID: L-DOPA induced dyskinesia

L-DOPA: Levodopa, 3,4-dihydroxy-L-phenylalanine

MGI: Mouse Genome Informatics

MPTP: 1-methyl-4-phenyl-1,2,3,6 tetrahydropyridine

PD: Parkinson’s disease

SC: subcutaneous

Sid = once daily

PO: per os

IM=intramuscular

IP: intraperitoneal

IC: intracarotid

UPDRS: Unified Parkinson's Disease Rating Scale

MFB: medial forebrain bundle

NHP: non-human primate

SN: substantia nigra

TCD = Total cumulative dose

Tid = three times daily

**Definitions**

*Approval status*: Whether a therapy has been approved for clinical use in Parkinsons Disease

*Intervention*: Introduction of variable (pharmaceutical, phytochemical, physical, genetic, behavioral or environmental) into a model system with the intent to assess its effect upon outcome.

*Mechanism of action of an intervention:* Means by which an intervention influences a biological system such as a mechanism of action of a drug, effect on a cellular pathway or a physiologic function.

*Model:* An animal system harboring specific PD-related alleles, or an animal in which some mechanistic aspect of PD is modeled (e.g. MPTP intoxication).

*Outcome:* Effect of an intervention on overall disease severity in a patient population or animal model system, as defined by the authors in the abstract.

*Outcome measure:* A measurable clinical or non-clinical parameter used to assess the effect of the intervention on the Parkinsonian phenotype

*Strain*: the (rodent) strain upon which the mutant allele resides (e.g. C57BL/6J).
