# Supplementary material for: Established patterns of animal study design undermine translation of disease-modifying therapies for Parkinson’s disease
Source: PLoS One. 2017 Feb 9;12(2):e0171790. doi: 10.1371/journal.pone.0171790 (PMC5300282; doi:10.1371/journal.pone.0171790)
Supplement: S2 Appendix — (DOCX) [file pone.0171790.s002.docx]

| Rat | PMID:24939694 | Mouse | PMID:23077045 \| PMCID:PMC3501652 | NHP | PMID:16885224 | |  |  |
| --- | --- | --- | --- | --- | --- | --- | --- | --- |
| Rat | PMID:25120226 | Mouse | PMID:23178948 | NHP | PMID:22728661 | |  |  |
| Rat | PMID:25123753 | Mouse | PMID:24786396 \| PMCID:PMC4007078 | NHP | PMID:24744393 \| PMCID:PMC4039459 | | | |
| Rat | PMID:25159816 | Mouse | PMID:24909656 | NHP | PMID:17586681 \| PMCID:PMC1896134 | | | |
| Rat | PMID:25432430 | Mouse | PMID:25491263 | NHP | PMID:17920901 \| PMCID:PMC2174366 | | | |
| Rat | PMID:25446857 | Mouse | PMID:25541039 | NHP | PMID:18394605 \| PMCID:PMC2483423 | | | |
| Rat | PMID:25528406 | Mouse | PMID:25626558 | NHP | PMID:22815935 \| PMCID:PMC3398927 | | | |
| Rat | PMID:25692070 \| PMCID:PMC4322303 | Mouse | PMID:25637482 | NHP | PMID:23562290 | |  |  |
| Rat | PMID:25810874 \| PMCID:PMC4366741 | Mouse | PMID:25680233 | NHP | PMID:23913185 \| PMCID:PMC3863793 | | | |
| Rat | PMID:25815475 \| PMCID:PMC4376720 | Mouse | PMID:25702135 | NHP | PMID:2871381 | |  |  |
| Rat | PMID:25862572 \| PMCID:PMC4457638 | Mouse | PMID:25724264 | NHP | PMID:16464239 | |  |  |
| Rat | PMID:25897205 \| PMCID:PMC4396575 | Mouse | PMID:25769424 | NHP | PMID:18222654 | |  |  |
| Rat | PMID:25914172 | Mouse | PMID:25798108 \| PMCID:PMC4351630 | NHP | PMID:18657577 | |  |  |
| Rat | PMID:25918669 \| PMCID:PMC4396568 | Mouse | PMID:25825522 \| PMCID:PMC4420923 | NHP | PMID:17879087 | |  |  |
| Rat | PMID:25999296 | Mouse | PMID:25883632 \| PMCID:PMC4392681 | NHP | PMID:22178201 | |  |  |
| Rat | PMID:26001615 | Mouse | PMID:25883828 \| PMCID:PMC4390107 | NHP | PMID:16532454 | |  |  |
| Rat | PMID:26013581 \| PMCID:PMC4506434 | Mouse | PMID:25983275 | NHP | PMID:16772809 | |  |  |
| Rat | PMID:26119304 | Mouse | PMID:26006269 | NHP | PMID:16925991 | |  |  |
| Rat | PMID:26146528 \| PMCID:PMC4469843 | Mouse | PMID:26051278 | NHP | PMID:16991143 | |  |  |
| Rat | PMID:26167188 \| PMCID:PMC4488543 | Mouse | PMID:26051538 | NHP | PMID:17351422 | |  |  |
| Rat | PMID:26198255 \| PMCID:PMC4511027 | Mouse | PMID:26157004 \| PMCID:PMC4495236 | NHP | PMID:17373723 | |  |  |
| Rat | PMID:26282118 | Mouse | PMID:26188143 | NHP | PMID:18321484 | |  |  |
| Rat | PMID:26470809 | Mouse | PMID:26210618 | NHP | PMID:18759357 | |  |  |
| Rat | PMID:26480869 | Mouse | PMID:26255701 \| PMCID:PMC4530460 | NHP | PMID:19619533 | |  |  |
| Rat | PMID:26481946 \| PMCID:PMC4615887 | Mouse | PMID:26399293 | NHP | PMID:20108359 | |  |  |
| Rat | PMID:26491600 \| PMCID:PMC4603313 | Mouse | PMID:26405176 \| PMCID:PMC4600625 | NHP | PMID:20303948 | |  |  |
| Rat | PMID:26101828 | Mouse | PMID:26434409 | NHP | PMID:20434890 | |  |  |
| Rat | PMID:26490873 | Mouse | PMID:26453752 | NHP | PMID:20721904 | |  |  |
| Rat | PMID:24628580 | Mouse | PMID:26453962 | NHP | PMID:20843474 | |  |  |
| Rat | PMID:26124091 \| PMCID:PMC4507186 | Mouse | PMID:26523792 | NHP | PMID:20973483 | |  |  |
| Rat | PMID:24782287 | Mouse | PMID:26538809 \| PMCID:PMC4604129 | NHP | PMID:21562283 | |  |  |
| Rat | PMID:25261754 \| PMCID:PMC4262739 | Mouse | PMID:23391521 \| PMCID:PMC3604118 | NHP | PMID:21953539 | |  |  |
| Rat | PMID:25267343 \| PMCID:PMC4330501 | Mouse | PMID:25128026 | NHP | PMID:23029119 \| PMCID:PMC3447761 | | | |
| Rat | PMID:25499799 | Mouse | PMID:25608039 \| PMCID:PMC4301865 | NHP | PMID:24447715 | |  |  |
| Rat | PMID:25693197 \| PMCID:PMC4332861 | Mouse | PMID:25907448 | NHP | PMID:24769173 | |  |  |
| Rat | PMID:25697393 | Mouse | PMID:25980996 | NHP | PMID:26415982 | |  |  |
| Rat | PMID:25711470 | Mouse | PMID:26517532 | NHP | PMID:16940766 | |  |  |
| Rat | PMID:25749357 \| PMCID:PMC4466038 | Mouse | PMID:23169921 \| PMCID:PMC3501971 | NHP | PMID:22901956 | |  |  |
| Rat | PMID:25962878 | Mouse | PMID:23409128 \| PMCID:PMC3567051 | NHP | PMID:23504289 | |  |  |
| Rat | PMID:26119238 | Mouse | PMID:24697290 \| PMCID:PMC4206694 | NHP | PMID:25449794 | |  |  |
| Rat | PMID:26195164 \| PMCID:PMC4623086 | Mouse | PMID:24804730 \| PMCID:PMC4013129 | NHP | PMID:23389842 | |  |  |
| Rat | PMID:26207892 | Mouse | PMID:25106480 | NHP | PMID:23402994 | |  |  |
| Rat | PMID:26217192 | Mouse | PMID:25193021 | NHP | PMID:23756168 | |  |  |
| Rat | PMID:26222442 \| PMCID:PMC4519335 | Mouse | PMID:25301748 | NHP | PMID:23770260 | |  |  |
|  |  | Mouse | PMID:25581060 | NHP | PMID:24135129 | |  |  |
|  |  | Mouse | PMID:25677261 | NHP | PMID:24456747 | |  |  |
|  |  | Mouse | PMID:25702964 | NHP | PMID:24520383 \| PMCID:PMC3919785 | | | |
|  |  | Mouse | PMID:25770828 | NHP | PMID:24572591 | |  |  |
|  |  | Mouse | PMID:25857436 | NHP | PMID:24610195 | |  |  |
|  |  | Mouse | PMID:25895692 | NHP | PMID:24865335 | |  |  |
|  |  | Mouse | PMID:25929185 | NHP | PMID:24990932 \| PMCID:PMC4078088 | | | |
|  |  | Mouse | PMID:25929279 | NHP | PMID:25046277 | |  |  |
|  |  | Mouse | PMID:25994206 \| PMCID:PMC4438574 | NHP | PMID:25820831 | |  |  |
|  |  | Mouse | PMID:26006268 | NHP | PMID:25907446 | |  |  |
|  |  | Mouse | PMID:26028469 | NHP | PMID:26071982 | |  |  |
|  |  | Mouse | PMID:26045688 \| PMCID:PMC4452523 | NHP | PMID:26456231 | |  |  |
|  |  | Mouse | PMID:26104567 | NHP | PMID:22766137 | |  |  |
|  |  | Mouse | PMID:26108182 | NHP | PMID:23300984 \| PMCID:PMC3536787 | | | |
|  |  | Mouse | PMID:26114390 | NHP | PMID:23403361 | |  |  |
|  |  | Mouse | PMID:26164453 | NHP | PMID:23594934 \| PMCID:PMC3728183 | | | |
|  |  | Mouse | PMID:26314634 \| PMCID:PMC4594956 | NHP | PMID:23724014 \| PMCID:PMC3665802 | | | |
|  |  | Mouse | PMID:26522450 | NHP | PMID:24002224 | |  |  |
|  |  | Mouse | PMID:26545632 | NHP | PMID:25043598 \| PMCID:PMC4165715 | | | |
|  |  | Mouse | PMID:25716193 \| PMCID:PMC4407187 | NHP | PMID:25498223 | |  |  |
|  |  | Mouse | PMID:23295906 \| PMCID:PMC3645298 | NHP | PMID:25559284 | |  |  |
|  |  | Mouse | PMID:23512787 | NHP | PMID:25771209 | |  |  |
|  |  | Mouse | PMID:25406165 \| PMCID:PMC4306720 | NHP | PMID:26373985 | |  |  |
|  |  | Mouse | PMID:25908255 | NHP | PMID:16885224 | |  |  |
|  |  | Mouse | PMID:26013581 \| PMCID:PMC4506434 | NHP | PMID:12810526 | |  |  |
|  |  | Mouse | PMID:26141845 | NHP | PMID:18346734 \| PMCID:PMC2855210 | | | |
|  |  | Mouse | PMID:26282118 | NHP | PMID:1893983 | |  |  |
|  |  | Mouse | PMID:26302060 | NHP | PMID:19399891 \| PMCID:PMC2881694 | | | |
|  |  | Mouse | PMID:26453833 | NHP | PMID:22810218 | |  |  |
|  |  | Mouse | PMID:26477638 | NHP | PMID:24610674 | |  |  |
|  |  | Mouse | PMID:26187689 | NHP | PMID:25839189 | |  |  |
|  |  | Mouse | PMID:26409043 | NHP | PMID:8689042 | |  |  |
|  |  | Mouse | PMID:23145024 \| PMCID:PMC3492133 | NHP | PMID:22017994 | |  |  |
|  |  | Mouse | PMID:24629674 | NHP | PMID:2402638 | |  |  |
|  |  | Mouse | PMID:25263579 \| PMCID:PMC4415848 | NHP | PMID:12223537 | |  |  |
|  |  | Mouse | PMID:26292069 |  |  |  |  |  |
|  |  | Mouse | PMID:26349993 |  |  |  |  |  |
|  |  | Mouse | PMID:25943481 |  |  |  |  |  |
|  |  | Mouse | PMID:24041971 |  |  |  |  |  |
|  |  | Mouse | PMID:24412329 |  |  |  |  |  |
|  |  | Mouse | PMID:25678053 |  |  |  |  |  |
|  |  | Mouse | PMID:25857256 |  |  |  |  |  |
|  |  | Mouse | PMID:26111725 |  |  |  |  |  |
|  |  | Mouse | PMID:25499799 |  |  |  |  |  |
|  |  | Mouse | PMID:25640758 |  |  |  |  |  |
|  |  | Mouse | PMID:25711470 |  |  |  |  |  |
|  |  | Mouse | PMID:25983633 \| PMCID:PMC4415566 |  |  |  |  |  |
|  |  | Mouse | PMID:26499517 \| PMCID:PMC4620555 |  |  |  |  |  |
