# Supplementary material for: Established patterns of animal study design undermine translation of disease-modifying therapies for Parkinson’s disease
Source: PLoS One. 2017 Feb 9;12(2):e0171790. doi: 10.1371/journal.pone.0171790 (PMC5300282; doi:10.1371/journal.pone.0171790)
Supplement: S1 Table — (DOCX) [file pone.0171790.s004.docx]

**S1 Table: Studies in animal models of PD prior to first report in humans (PubMed)**

|  | **Human: earliest report of use in humans (PMID)** | | **Animal: earliest report of use in a model of PD, prior to first human report (PMID)** | | | | |
| --- | --- | --- | --- | --- | --- | --- | --- |
|  | PubMed (PMID) | FDA or other approval | Mouse | Rat | Macaque | Vervet | Marmoset |
| **Dopamine receptor agonists** | | | | | | | |
| L-Dopa (D1-D5) | 1966 (5327616) | 1970 | 0 | 0 | 0 | 0 | 0 |
| Lisuride (D2-5; 5HT) | 1979 (91855) | No  (Approved in EU, UK, China) | 0 | 593443  307642  156312 | 0 | 0 | 0 |
| Pergolide | 1979 (91856) | 1988; Withdrawn 2007 | 0 | 0 | 0 | 0 | 0 |
| Apomorphine (D1/D2 agonist) | 1951 (14913646) | 2004 | 0 | 0 | 0 | 0 | 0 |
| Bromocryptine (D2 agonist; various 5 HT) | 1974 (4425916) | 1978 | 0 | 4146398 | 0 | 0 | 0 |
| Rotigotine (D2-5 agonist) | 2001 (11391739) | 2007 | 2566677 | 3130270  7910948 | 7910948  9205801 | 0 | 2572426 |
| Ropinirole | 1989 (2567448) | 1997 | 0 | 0 | 0 | 0 | 0 |
| Pramipexole | 1992 (1350237) | 1997 | 0 | 0 | 0 | 0 | 0 |
| **Monoamine oxidase inhibitors** | | | | | | | |
| Rasagiline | 2000 (11575866) | 2006 | 3935467 | 9564629 | 0 | 0 | 0 |
| Selegiline | 1978 (370348) | 2006 | 0 | 0 | 0 | 0 | 0 |
| **Catechol-O-methyltransferase inhibitors** | | | | | | | |
| Entacapone | 1993 (8112370) | 2003 | 0 | 1300941  8119326 | 0 | 0 | 0 |
| Tolcapone | 1993 (8099689) | 1998 | 0 | 0 | 0 | 0 | 0 |
| **Adenosine A2a Receptor Antagonists** | | | | | | | |
| Istradefylline | 2003 (12913186) | 2013 (Japan) | 10591873  10908627  11319241  11902116 | 10996458  11090641  11902116 | 10331698 | 0 | 9546333  9760134  10739638 |
| **Deep brain stimulation (DBS)** | | | | | | | |
| Subthalamic nuclear stimulation |  | 2002 | 0 | 0 | 2402638  1758446  2011750  1361741  8261116 | 0 | 0 |

Dates of the earliest published human studies for each intervention were obtained from PubMed ((<http://www.ncbi.nlm.nih.gov/pubmed>). To determine whether animal studies for each drug had appeared prior to studies in humans, dates of the earliest published reports for each intervention in each of 5 species (mouse, rat, marmoset, vervet monkey and macaque) were obtained from PubMed. Search strategy utilized the following filters:

“*” AND Parkinson’s disease, limited by Clinical Trials

“*” AND Parkinson’s disease AND “**”

* Intervention name

** Animal species (Mouse, Rat, Vervet monkey, Macaque, Marmoset)

These included studies of any type, including pharmacologic or basic mechanistic studies, and did not require the animal study to be performed in a model of PD. The entire list of animal studies published prior to publication of a human study was then examined to extract those reports in which efficacy had been tested using an animal model of PD.
