# Supplementary material for: Established patterns of animal study design undermine translation of disease-modifying therapies for Parkinson’s disease
Source: PLoS One. 2017 Feb 9;12(2):e0171790. doi: 10.1371/journal.pone.0171790 (PMC5300282; doi:10.1371/journal.pone.0171790)
Supplement: S2 Table — (DOCX) [file pone.0171790.s005.docx]

**S2 Table: Methods of animal model studies for drugs approved for PD, performed prior to approval date (PubMed; n=28*)**

| **1. Istradefylline (Adenosine A2A antagonist)** | | | | | | | | |
| --- | --- | --- | --- | --- | --- | --- | --- | --- |
|  | | | **Model details** | | | **Outcome** | | |
| **PMID;year** | | **Species** | **Model** | **Protocol** | **Timing of intervention** | **Non clinical** | **Clinical *** | |
| 10591873; 1999 | | Mouse | Reserpine or haloperidol-induced catalepsy | Reserpine SQ 5mg/kg  Haloperidol IP 1mg/kg | 18-24 hours after reserpine or haloperidol administration | NR | Improved | |
| 10591873; 1999 | | Mouse | MPTP | MPTP IP 30mg/kg once daily for 5 days | 30 min after final dose of MPTP | NR | Improved | |
| 10908627; 2000 | | Mouse | D2R -/- mice | Knockout, Baik 1995 | KO and WT, unknown age and gender | Normalized striatal expression of enkephalin and substance P | Improved | |
| 11319241; 2001 | | Mouse | MPTP | 20-40 mg/kg IP once;  20 mg/kg IP, 4X, every 2hours | 10 minutes prior to MPTP injection | Attenuated loss of striatal dopamine | NR | |
| 11902116; 2002 | | Mouse | MPTP | 40 mg/kg IP once | 1 hour after MPTP injection; neuroprotection lost if given more than 5 hours after injection | Protected striatal neurons; reduced gliosis | NR | |
| 11902116; 2002 | | Rat | 6-OHDA | Unilateral injection of 7µg 6-OHDA into striatum | Once daily 7 days, first dose 50 min before 6-OHDA | Protected against the loss of nigral dopaminergic neuronal cells | NR | |
| 10996458; 2000 | | Rat | 6-OHDA | Unilateral injection of 8 µg 6-OHDA into MFB. Tested with apomorphine 0.1 mg/kg, SC 6 days later. | 2 weeks after 6-OHDA | Decreased GABA levels (globus pallidus) | NR | |
| 11090641; 2000 | | Rat | 6-OHDA | Unilateral injection of 8 µg 6-OHDA into MFB. Tested with apomorphine 0.1 mg/kg, SC 6 days later. | 2 weeks after 6-OHDA | NR | Improved | |
| 10331698; 1999 | | Macaque | MPTP/LID | 2-3 mg/animal SC 1X/week, until stable Parkinsonism achieved (1-44 weeks). LID induced (L-DOPA/benserazide) over several months. | Months after MPTP/L-DOPA, (once stable LID achieved; varied by animal) | NR | Improved | |
| 9546333; 1998 | | Marmoset | MPTP/LID | 2 mg/kg SC daily for 5 days  6 to 8 weeks recovery period. LID induced (L-DOPA/benserazide) for 21 days. | 7 weeks to up to 8 months (once stable LID achieved) | NR | Improved | |
| 9760134; 1998 | | Marmoset | MPTP  /LID | 2 mg/kg SC daily for 5 days  6 to 8 weeks recovery period. LID induced (L-DOPA/benserazide) for 21 days. | 7 weeks to up to 8 months (once stable LID achieved) | NR | Improved | |
| 10739638; 2000 | | Marmoset | MPTP/LID | 2 mg/kg SC daily for 5 days  6 to 8 weeks recovery period. LID induced (L-DOPA/benserazide) for 21 days. | 7 weeks to up to 8 months (once stable LID achieved) | NR | Improved | |
| **2. Rotigotine (predominantly D2-like and D5 receptor agonist)** | | | | | | | | |
|  | |  | **Model details** | | | **Outcome** | | |
| **PMID;year** | | **Species** | **Model** | **Protocol** | **Timing of intervention** | **Non clinical** | | **Clinical **** |
| 2566677; 1989 | | Mouse | 6-OHDA | Unilateral injection of 100 nmol of 6-OHDA into striatum. 7 days later, turning activity with dopamine agonist | At least 1 week later | D1/D2 receptor occupancy | | Improved |
| 3130270; 1988 | | Rat | 6-OHDA | Unilateral injection of 8 µg 6-OHDA into substantia nigra. Apomorphine 0.5 mg/kg IP after 10 and 20 days to establish rotational behavior. | At least one month later | Inhibited release of dopamine and acetylcholine from rabbit striatal slices | | Improved |
| 7910948; 1994 | | Rat | 6-OHDA | Unilateral injection of 32 ug 6-OHDA into substantia nigra. 7 days later, turning activity in response to apomorphine examined. | At least 2 week later | Dopamine receptor binding assays, dopamine turnover | | Improved |
| 7910948; 1994 | | Macaque | MPTP | Unilateral intracarotid injection, 3mg MPTP/ Hemiparkinsonian model | After MPTP, following stable hemiparkinsonism, time period not stated | Recognizes the high and low affinity states of the D2 receptor | | Improved |
| 9205801; 1997 | | Macaque | MPTP | Unilateral intracarotid injection, 3mg MPTP/ Hemiparkinsonian model | After MPTP, following stable hemiparkinsonism, time period not stated | NR | | Improved |
| 2572426; 1989 | | Marmoset | MPTP | 9 day protocol: 1-4 mg/kg IP daily  6 day protocol: 2 mg/kg SC daily | 9 day protocol: At least 6 weeks later  6 day protocol: Several weeks later | NR | | Improved |
| **3. Rasagiline (Monoamine oxidase B inhibitor)** | | | | | | | | |
|  | | | **Model details** | | | **Outcome** | | |
| **PMID;year** | **Species** | | **Model** | **Protocol** | **Timing of intervention** | **Non clinical** | | **Clinical **** |
| 3935467; 1985 | | Mouse | MPTP | 20 mg/kg IP, 4X, every 2hours | One day prior to MPTP | Protected against MPTP-induced decline in striatal dopamine | | NR |
| 9564629; 1998 | | Rat | alpha-methyl-p-tyrosine (alpha-MpT)-induced hypokinesia | alpha-MpT 100-120mg/kg IP | Immediately prior to alpha-MpT | NR | | Improved |
| 9564629; 1998 | | Rat | Haloperidol -induced badykinesia | Haloperidol 1.5mg/kg SC | 2 hours prior to haloperidol | NR | | Improved |
| **4. Entacapone (Catechol-O-methyltransferase COMT inhibitor)** | | | | | | | | |
|  | | | **Model details** | | | **Outcome** | | |
| **PMID;year** | | **Species** | **Model** | **Protocol** | **Timing of intervention** | **Non clinical** | | **Clinical **** |
| 8119326; 1993 | | Rat | 6-OHDA | 6-OHDA 8µg in MFB unilateral  2 weeks later: 0.05mg/kg apomorphine | 3 weeks after 6-OHDA | NR | | Improved |
| **5. Lisuride (predominantly D2-like agonist; partial agonist for several serotonin receptors; antagonist at the serotonin 5-HT2B receptor)** | | | | | | | | |
|  | | | **Model details** | | | **Outcome** | | |
| **PMID;year** | | **Species** | **Model** | **Protocol** | **Timing of intervention** | **Non clinical** | | **Clinical **** |
| 593443; 1977 | | Rat | 6-aminonicotina-mide (6-AN) and reserpine induced rigidity | 10 mg 6-AN IP once  5mg/kg reserpine IP | Months after 6-AN  One hour after reserpine | NR | | Improved |
| 307642; 1978 | | Rat | 6-OHDA | 6-OHDA 3.5 µg in right MFB  10-15d later: apomorphine 1 mg kg IP | 2-3 weeks after 6-OHDA | NR | | Improved |
| 307642; 1978 | | Rat | 5,6_dihydroxytryptamine (5,6-DHT) | 5,6-DHT 10 pg in right MFB  10-15d later: apomorphine 1 mg kg IP | 2-3 weeks after 5,6 DHT | NR | | Improved |
| 307642; 1978 | | Rat | prochlorperazine methanesulfonate catalepsy | Prochlorperazine 15 mg/kg IP | 3-6 days later | NR | | Improved |
| 156312; 1979 | | Rat | 6-aminonicotina-mide (6-AN) and reserpine induced rigidity | 10 mg 6-AN IP once  5mg/kg reserpine IP | Following 6-AN and reserpine, time not stated | Catecholamine turnover | | Improved |
| **6. Bromocryptine (agonist at monoamine receptors; predominantly an agonist of dopamine D2 receptors)** | | | | | | | | |
|  | | | **Model details** | | | **Outcome** | | |
| **PMID;year** | | **Species** | **Model** | **Protocol** | **Timing of intervention** | **Non clinical** | | **Clinical **** |
| 4146398; 1973 | | Rat | 6-OHDA | 6-OHDA 8µg, substantia nigra, Ungerstedt 1968 | Following 6-OHDA, time not stated | Catecholamine turnover | | Improved |
| 156312; 1979 | | Rat | 6-aminonicotina-mide (6-AN) and reserpine induced rigidity | 10 mg 6-AN IP once  5mg/kg reserpine IP | Following 6-AN and reserpine, time not stated | Catecholamine turnover | | Improved |
| **7. Deep brain stimulation** | | | | | | | | |
|  | | | **Model details** | | | **Outcome** | | |
| **PMID;year** | | **Species** | **Model** | **Protocol** | **Timing of intervention** | **Non clinical** | | **Clinical **** |
| 2402638; 1990 | | Macaque | MPTP | Unilateral intracarotid injection, 3mg MPTP/ Hemiparkinsonian model | Following MPTP; Unilateral lesion of the subthalamic nucleus with ibotenic acid | NR | | Improved |
| 1758446;1991 | | Macaque | MPTP | Unilateral intracarotid injection, 3mg MPTP/ Hemiparkinsonian model | Following MPTP; Unilateral lesion of the subthalamic nucleus | NR | | Improved |
| 2011750; 1991 | | Macaque | MPTP | Unilateral intracarotid injection, 3mg MPTP/ Hemiparkinsonian model | Following MPTP; Unilateral lesion of the GPi (inadvertent) | NR | | Improved |
| 1361741; 1992 | | Macaque | MPTP | Unilateral intracarotid injection, 3mg MPTP/ Hemiparkinsonian model | Following MPTP; Thermocoagulative lesions of the STN | NR | | Improved |
| 8261116; 1993 | | Macaque | MPTP | Unilateral intracarotid injection, 3mg MPTP/ Hemiparkinsonian model | Following MPTP; Subthalamic high-frequency stimulation | NR | | Improved |

* Methodologic details of 28 preclinical publications in animal models of PD – one publication is defined as a unique PMID. Several publications utilized more than one species or contained more than experiment.

** Definition of improved clinical outcomes for various models

1. MPTP model: ameliorates hypolocomotion and bradykinesia.

2. MPTP/LID model: ameliorates hypokinetic Parkinsonian motor phenotype, reduces L-dopa-induced dyskinesia (LID).

3. MPTP/Hemiparkinsonian model: amelioration of MPTP induced contralateral limb dystonic postures, rigidity, bradykinesia and reduced fine motor ability.

4. 6-OHDA model: 6-OHDA induces ipsilateral rotational behavior; dopamine agonists (L-DOPA, apomorphine) induce contralateral rotation. An improved outcome for an intervention is defined as prolongation of turning duration beyond that seen with dopamine agonists.

5. 5,6_dihydroxytryptamine (5,6-DHT): As for 6-OHDA model.

6. Alpha-methyl-p-tyrosine (alpha-MpT)-induced hypokinesia: ameliorated hypokinesia

7. 6-Aminonicotinamide (6-AN) model: gastrocnemius muscle rigidity quantified by electrical recording.

8. Prochlorperazine catalepsy model: ameliorated catalepsy.

9. Reserpine and haloperidol-induced catalepsy: reversal of catalepsy and rigidity; gastrocnemius muscle rigidity quantified by electrical recording.

10. Dopamine 2 Receptor (D2R) -/- mice: rescued behavioral parameters , rotarod, open field , ring test .
