# Supplementary material for: Established patterns of animal study design undermine translation of disease-modifying therapies for Parkinson’s disease
Source: PLoS One. 2017 Feb 9;12(2):e0171790. doi: 10.1371/journal.pone.0171790 (PMC5300282; doi:10.1371/journal.pone.0171790)
Supplement: S3 Table — (DOCX) [file pone.0171790.s006.docx]

**S3 Table: Comparison of clinical trial and preclinical data for compounds failing to achieve comparable results across humans and animals PubMed; n=70*).**

| **1. Creatine (supports mitochondrial phosphocreatine and ATP production)** | | | | | | | | | | | |
| --- | --- | --- | --- | --- | --- | --- | --- | --- | --- | --- | --- |
|  | | **Model/Patient details** | | | | | | | | **Outcome** | |
| **PMID; year** | **Species** | **Model/Patient** | | | | **Intervention** | | | **Timing of intervention** | **Non clinical** | **Clinical** |
| 25668262; 2015 | Human | PD | | | | Creatine 10 g/day for 5-8 years | | | Early untreated and treated PD | NR | No effect:  I^o^ OM: difference in clinical decline from baseline to 5-year follow-up. |
| 17030762;  2006 | Human | PD | | | | Creatine 20 g/day for 6 days, then 2 g daily for 6 months and 4 g daily for 2 years. | | | PD patients on standard symptomatic therapy | No effect: dopamine transporter SPECT. | Mixed effect:  No effect on overall UPDRS but smaller dose increase of dopaminergic therapy needed |
| 19476553; 2009 | Mouse | MPTP 40mg/kg daily SQ for 28 days | | | | 2% creatine-enriched diet | | | Began 1 week prior to MPTP | Improved | NR |
| 14645986;  2003 | Mouse | MPTP 20 mg/kg IP every 2 hours, 4X | | | | 2% creatine-enriched diet | | | Began 1 week prior to MPTP | Improved | NR |
| 10222117;  1999 | Mouse | MPTP 15 mg/kg IP every 2 hours, 5 X | | | | 1% creatine-enriched diet | | | Began 2 weeks prior to MPTP | Improved | NR |
| 19476553; 2009 | Rat | 3-NP 50mg/kg daily for 7 days | | | | 2% creatine-enriched diet | | | Began 1 week prior to 3-NP | Improved | NR |
| 18762218; 2009 | Rat | 6-OHDA 6 μg IC injection unilateral MFB, followed by L-DOPA 32 days later. L-DOPA given for 21 days | | | | 2% creatine-enriched diet | | | Began 3 weeks after 6-OHDA, 10 days prior to L-DOPA | Improved | Mixed effect: Improved on AIMS scale but not cylinder test. |
| **2. Mitoquinone (MitoQ; Coenzyme Q10 analogue)** | | | | | | | | | | | |
|  |  | **Model/Patient details** | | | | | | | | **Outcome** | |
| **PMID; year** | **Species** | **Model/Patient** | | | **Intervention** | | | | **Timing of intervention** | **Non clinical** | **Clinical *** |
| 20568096;  2010 | Human | PD | | | MitoQ 40 mg or 80 mg for 12 months | | | | Early PD, before initiation of symptomatic therapy. | NR | No effect: I^o^ OM: total UPDRS score |
| 20828611; 2010 | Mouse | MPTP 25 mg/kg IP once daily for 5 days | | | MitoQ 4 mg/kg | | | | Began 1 day before MPTP, until 7 days after MPTP | Improved | Improved: Open field and rotarod tested 5 days after last MPTP injection |
| **3. Coenzyme Q (electron acceptor in mitochondrial complexes I and II)** | | | | | | | | | | | |
|  | | **Model/Patient details** | | | | | | | | **Outcome** | |
| **PMID; year** | **Species** | **Model/Patient** | | | | | | **Intervention** | **Timing of intervention** | **Non clinical** | **Clinical** |
| 24664227;  2014 | Human | PD | | | | | | CoQ_10_ 200 or 2400 mg/day and vitamin E 1200 IU/day for 16m | Early PD, using symptomatic PD therapy 90 days or less | NR | No effect: I^o^ OM: total UPDRS score (Parts I-III) |
| 24410614; 2014 | Human | PD | | | | | | CoQ_10_ 400, 800, 1200, and 2400 mg/day for 2 weeks | Early PD | Biomarkers of oxidative damage; baseline ubiquinol | Improved 2400mg/day  I^o^ OM: total UPDRS score |
| 17502459;  2007 | Human | PD | | | | | | CoQ_10_ 100 mg three times daily for 3 months | Midstage PD, without motor fluctuations | Plasma CoQ_10_ | No effect: I^o^ OM: total UPDRS score (Parts I-III) |
| 12697283;  2003 | Human | PD | | | | | | CoQ_10_  180 mg twice daily for 1 month | Midstage PD, without motor fluctuations | NR | Improved: I^o^ OM: total UPDRS score. Motor symptoms did not improve |
| 12374491;  2002 | Human | PD | | | | | | CoQ_10_ 300, 600, or 1200 mg/day | Early PD, before initiation of symptomatic therapy | NR | Improved (1200mg/day)  I^o^ OM: total UPDRS score |
| 19476553;  2009 | Mouse | MPTP SQ 40mg/kg daily for 28 days | | | | | | 1% CoQ_10_ diet | Began 1 week prior to MPTP | Improved | NR |
| 17973981;  2008 | Mouse | Acute: MPTP 10 mg/kg IP q2 hours, 3X  Subacute: MPTP 10 mg/kg IP daily for 4 days  Chronic: MPTP SQ 40mg/kg/day for 28 days | | | | | | CoQ_10_ 1600 mg/kg/day | Acute: 2m – 2w before MPTP through 1m – 1w after last dose  Subacute: 3m before MPTP until 2w after last dose  Chronic: 1m before MPTP until 1m after last dose | Improved | NR |
| 9479058;  1998 | Mouse | MPTP 15 mg/kg IP q2hrs 5X | | | | | | CoQ_10_ 200 mg/kg/day | Began 1 month prior to MPTP, until 1 week after last dose. | Improved | NR |
| 24775711;  2014 | Mouse | MPTP 25 mg/kg IP once daily for 5 days | | | | | | CoQ_10_ 30 mg/kg/day | Prophylactic: Began 2 weeks before MPTP, continue for duration  Therapeutic: Began on last day of MPTP, continue for duration | Improved | Improved: Beam walk test |
| 24483602;  2014 | Rat | Paraquat 10 mg/kg IP every 5 days, 4X | | | | | | 50 mg CoQ_10_/ml | Began on last day of PQ, continue for 8 weeks | Improved | Improved: Beam walk test |
| 18817789;  2008 | Rat | Rotenone 2.5 mg/kg/day IP for 60 days | | | | | | CoQ_10_ 200-600 mg/kg/day | Began on day 20, continue through 60 days | Improved | Improved: Catalepsy test |
| 12810526  2013 | Vervet | MPTP 1.5 mg/kg cumulative, im, over 5 days | | | | | | 100 mg CoQ10 | Prophylactic: Began 10 days before MPTP | Improved | NR |
| **4. Pioglitazone (Peroxisome proliferator-activated receptor Υ agonist)** | | | | | | | | | | | |
|  | | | **Model/Patient details** | | | | | | | **Outcome** | |
| **PMID** | **Species** | | **Model/Patient** | | | | | **Intervention** | **Timing of intervention** | **Non clinical** | **Clinical** |
| 26116315;  2015 | Human | | Early PD | | | | | 15 -45 mg/day pioglitazone for 44 weeks | Early PD, stable regimen of rasagiline or selegiline | NR | No effect  I^o^ OM: total UPDRS score |
| 22391246;  2012 | Mouse | | MPTP 20 mg/kg IP q2hrs 4X | | | | | Pioglitazone 50 mg/kg PO once daily for 14 days | Began 6 days before MPTP, continued for 7 days after MPTP | Improved | Improved: Actimetry |
| 18332857;  2008 | Mouse | | MPTP 30 mg/kg SQ once | | | | | Pioglitazone 20 mg/kg BID PO for 7 days | Began 7 days before MPTP | Improved | Improved: Beam walk test |
| 14690537;  2004 | Mouse | | MPTP 30 mg/kg IP daily for 2-5 days | | | | | Pioglitazone 20 mg/kg/day PO | Began 4 days before MPTP, given through last MPTP dose | Improved | NR |
| 12153485;  2002 | Mouse | | MPTP 15 mg/kg IP q2hrs 4X | | | | | Pioglitazone 20 mg/kg/day PO | Began 3 days before MPTP, given for 2-8 days | Improved | NR |
| 22391246;  2012 | Rat | | 6-OHDA 3 μg IC, bilateral, MFB | | | | | Pioglitazone 50 mg/kg PO once daily for 21 days | Began 6 days before 6-OHDA, continued throughout to day 15 | Improved | Improved: Grip test, actimetry |
| 25127682;  2014 | Rat | | MPTP 100 μg IC, bilateral injection | | | | | Pioglitazone 30 mg/kg PO for 5 days | Given for 5 days before MPTP | Improved | Improved: Open field, forced swim test, avoidance task |
| 22498097;  2012 | Rat | | 6-OHDA 12 μg IC, unilateral MFB injection | | | | | 1.Pioglitazone 20mg/kg PO for 14 days  2. Pioglitazone 20 mg/kg PO BID for 7 days  3. Pioglitazone 20 mg/kg PO BID for 7 days | 1. Began 7 days before 6-OHDA, continued for 7 days  2.Began 2 days after 6-OHDA, given until sac (for 7 more days)  3.Began 2 days after 6-OHDA, given for 7 more days. Sac after 7 more days | Improved | NR |
| 21600965;  2011 | Rat | | Rotenone 2.5 mg/kg SQ for 70 days | | | | | Pioglitazone (10 mg/kg, IP) and Retinoic acid (1 mg/kg, IP) | At the end of rotenone administration, treated for 15 days | Improved | Improved: Open field test |
| 20688106;  2011 | Rat | | MPTP 100 μg IC, substantia nigra injection | | | | | 1. Pioglitazone 5, 15 and 30 mg/kg PO  2. Pioglitazone 30 mg/kg PO for 22 days | 1. Began 1 hour after MPTP, sac 1 day later (acute)  2. Began 1 h after MPTP , continued through 22 days (chronic) | Improved | Acute: Improved at  15 and 30 mg/kg dose  Chronic: No effect  Open field test; Two-way avoidance task |
| 18983875; 2009 | Rat | | MPTP 100 μg IC, substantia nigra injection | | | | | Pioglitazone 10 and 30 mg/kg PO | Began 5 days before MPTP for 30 days | Improved | Improved: Morris water maze, passive avoidance task, |
| 18207323;  2008 | Rat | | Lipopolysaccharide, intrastriatal injection | | | | | Pioglitazone 20 mg/kg PO | Began 4 days prior to LPS | Improved | NR |
| 25559284;  2015 | Macaque | | MPTP mean cumulative dose 9.7 ± 6.3 mg, IV, once daily. Dyskinesia induced with L-DOPA/benserazide once stable Parkinsonism developed | | | | | Pioglitazone (3, 10, 30, and 60 mg/kg) PO | Given simultaneously with L-DOPA/benserazide | NR | Mixed effect:  Reduced duration of dyskinesia but reduced duration of L-DOPA anti-parkinsonian benefit |
| 21819568;  2011 | Macaque | | MPTP 3mg unilateral intracarotid injection 1X. | | | | | Pioglitazone 2.5-5 mg/kg for 3 months | Began 24 hours after MPTP | Improved | Improved:  Significant at 2/X time points (clinical rating scale), 1/X time point (fine motor skills) |
| **5. TCH 346/CGB3466B/omigapil (anti-apoptotic through GAPDH and SIAH inhibition)** | | | | | | | | | | | |
|  | | | **Model/Patient details** | | | | | | | **Outcome** | |
| **PMID** | **Species** | | **Model/Patient** | | | | | **Intervention** | **Timing of intervention** | **Non clinical** | **Clinical** |
| 17110281;2006 | Human | | PD | | | | | TCH 346 0.5, 2.5 or 10 mg/day for 12-18 months | Early PD, untreated | NR | No effect: I^o^ OM: time to requiring dopaminergic treatment. |
| 10971644;  2000 | Rat | | 6-OHDA 3 μg intracranial, bilateral | | | | | CGP 3466B 0.0014, 0.014, 0.14 or 1.4 mg/kg SQ | Two hours after 6-OHDA treatment | Improved except at highest dose, worsened at 1.4mg/kg | Improved except at highest dose |
| 14572443;  2003 | Macaque | | MPTP 2.5 mg left carotid artery. 8 weeks later, 1.25 mg MPTP right carotid artery | | | | | TCH346 0.014 mg/kg SQ BID for 14 days | 2 h after the second MPTP infusion | Prevented FDOPA uptake in the right striatum after second MPTP injection | Prevented worsening of motor symptoms after second MPTP injection. Did not reverse effects of the first MPTP treatment |
| 11205142; 2000 | Macaque | | MPTP 2.5 mg left carotid artery. 8 weeks later, 1.25 mg MPTP right carotid artery | | | | | TCH346 0.014 mg/kg SQ BID for 14 days | 2 h after the second MPTP infusion | NR | Prevented worsening of motor symptoms after second MPTP injection. |
| **6. CEP 1347 (anti-apoptotic effect through mixed lineage kinase inhibition)** | | | | | | | | | | | |
|  | | | **Model/Patient details** | | | | | | | **Outcome** | |
| **PMID** | **Species** | | **Model/Patient** | | | | | **Intervention** | **Timing of intervention** | **Non clinical** | **Clinical** |
| 17881719;  2007 | Human | | Early PD | | | | | CEP-1347 10, 25 or 50 mg BID, 21 months | Early PD, not yet requiring L-DOPA | No protection: Secondary; beta-CIT SPECT imaging of striatal dopamine transporters. | No effect: I^o^ OM: time to requiring dopaminergic treatment. |
| 10936203;  2000 | Mouse | | MPTP 40 mg/kg SQ once | | | | | CEP-1347 0.1 and 1.0 mg/kg | 4 h before MPTP | Improved | NR |
| 9918541; 1999 | Mouse | | MPTP 20 or 40 mg/kg SQ once | | | | | CEP-1347 10 ml/kg of 5% solution | 1. Began 4-6 days before MPTP for total 7 days  2. Began 7 days after MPTP, continued for 7 days | Improved | NR |
| 12516522;  2002 | Macaque | | MPTP 0.5 mg/kg once weekly for ten weeks or until predetermined functional deficit reached | | | | | CEP-1347 1 mg/kg | Prior to and simultaneous with MPTP | Improved | Improved: Attenuated loss of motor function |
| **7. Statins (HMG-CoA reductase inhibitors)** | | | | | | | | | | | |
|  | | | **Model/Patient details** | | | | | | | **Outcome** | |
| **PMID** | **Species** | | **Model/Patient** | | | | | **Intervention** | **Timing of intervention** | **Non clinical** | **Clinical** |
| 23283428; 2013 | Human | | PD | | | | | Simvastatin 40 mg/day: 2 treatments periods 10 days each separated by 4 days wash-out. | PD with dyskinesia | No effect:  phosphorylation in T and B-lymphocytes | No effect:  I^o^ OM: subjective discomfort caused by dyskinesia. |
| 15777746; 2005 | Mouse | | MPTP 15mg/kg IP q2hrs 4X | | | | | Simvastatin 10, 20, or 40 mg/kg/day PO for10 days | Began 5 days before MPTP | Improved | NR |
| 22789904;  2012 | Rat | | 6-OHDA 20 μg IC injection, unilateral striatum | | | | | Atorvastatin 10 and 20 mg/kg  Simvastatin 15 and 30 mg/kg | Began day after 6-OHDA for 14 days | Improved | Improved: Rotational activity, total locomotor activity |
| 21731633;  2011 | Rat | | 6-OHDA 32 μg IC unilateral injection MFB | | | | | Simvastatin 10 mg/kg/day | Prior to 6-OHDA, period not specified | Improved | Improved: Reduced anxiety: Elevated plus maze, |
| 15910782;  2005 | Rat | | 6-OHDA 32 μg IC unilateral injection MFB; rotational response measured 4 weeks later | | | | | Simvastatin 10 mg/kg/day for 4 weeks PO | Began 4 weeks after 6-OHDA | No change in striatal D1/D2 receptor expression due to OHDA or simvastatin. These measures did differ in prefrontal cortex | NR |
| 18434508;  2008 | Rat | | 6-OHDA 11 μg IC unilateral injection MFB  11 days later, l-DOPA /benserazide | | | | | Lovastatin 10 mg/kg IP | 3 days before L-DOPA initiated | Improved | Improved:  Abnormal involuntary movement scale |
| 23283428; 2013 | Macaque | | MPTP 0.2 mg/kg IV for 15 days; stable parkinsonism established after 8 weeks; followed by l-DOPA/ benserazide for 12 weeks to induce LID | | | | | Simvastatin (0, 1.5, 3 and 6 mg/kg) | Co-administration of levodopa and simvastatin | Target engagement:  Simvastatin 3 mg/kg reduced ERK1/2-induced phosphorylation in T and B cells | No effect: PD scores. Improved: 3 mg/kg simvastatin reduced dyskinesia scores |
| **8. Pardoprunox (SLV308; D(2/3) receptor partial agonist and full 5-HT(1A) receptor agonist)** | | | | | | | | | | | |
|  |  | | | **Model/Patient details** | | | | | | **Outcome** | |
| **PMID** | **Species** | | | **Model/Patient** | | | | **Intervention** | **Timing of intervention** | **Non clinical** | **Clinical** |
| 20198713;  2010 | Human | | | PD | | | | Pardoprunox 9-45 mg/day over 2 to 6 weeks and then maintained at this dose for a further 3 weeks | Early PD | NR | Improved.  I^o^ OM: UPDRS motor score  Nausea was reported by 32 of 68 (47.1%) |
| 21542016; 2011 | Human | | | PD | | | | Pardoprunox 6 mg/day; 12 mg/day or 12-42 mg/day | PD with UPDRS -Motor score ≥ 10 | NR | Improved.  I^o^ OM: UPDRS motor score 12-42 mg/day showed the highest dropout rate due to treatment-emergent adverse events |
| 22316635; 2012 | Human | | | PD | | | | Pardoprunox 42 mg/day | PD experiencing motor fluctuations. | NR | Decreased OFF time and increased ON time without troublesome dyskinesias.  High drop-out rate due to adverse events |
| 20434890; 2010 | Rat | | | 6-OHDA 10 µg intracranial, supranigral, once | | | | Pardoprunox 0.01, 0.03 and 0.10 mg/kg; PO, daily | After 10 days | NR | Induced contralateral turning behavior |
| 20434890; 2010 | Marmoset | | | MPTP 0.2 mg/kg/day SC for 5 days | | | | Pardoprunox 0.3 mg/kg; PO, daily | After 8-12 weeks | NR | Improved: increased locomotor activity, reduced motor disability |
| 20721904; 2010 | Marmoset | | | MPTP 0.2 mg/kg/day SC for 5 days | | | | Pardoprunox 0.1–0.3 mg/kg, PO, daily | After 6-8 weeks | NR | Improved; similar reduction of motor disability as L-DOPA, less dyskinesia |
| 20843474; 2010 | Marmoset | | | MPTP 0.2 mg/kg/day SC for 5 days  LID induced after 6-8 weeks | | | | Pardoprunox 0.0125 to 0.025 mg/kg, PO, daily | After 6-8 weeks | NR | Improved; similar reduction of motor disability as L-DOPA, less dyskinesia |
| **9. Preladenant (SCH 420814; selective adenosine A₂A receptor antagonist)** | | | | | | | | | | | |
|  |  | | | **Model/Patient details** | | | | | | **Outcome** | |
| **PMID** | **Species** | | | **Model/Patient** | | | **Intervention** | | **Timing of intervention** | **Non clinical** | **Clinical** |
| 23589371; 2013 | Human | | | PD | | | Preladenant 5 mg twice a day as a levodopa adjunct | | Moderate to severe PD taking levodopa | NR | I^o^ OM: adverse event - tolerated  2^o^ OM: Improved; sustained OFF time reductions and ON time increases |
| 21315654; 2011 | Human | | | PD | | | Preladenant 1, 2, 5, 10 mg/kg | | Moderate to severe PD taking levodopa | NR | I^o^ OM: mean daily off time reduced in 5, 10 mg/kg |
| 26523919; 2015 | Human | | | PD | | | Preladenant 1. 2 mg, 5 mg, or 10 mg BID  2. 2 mg or 5 mg BID | | Moderate to severe PD taking levodopa | NR | No effect  I^o^ OM: mean daily off time |
| 19332567; 2009 | Rat | | | 6-OHDA 8 μg intracranial, 1X. Followed by L-DOPA/ benserazide 2 weeks later | | | Preladenant 0.01–1 mg/kg SC | | Given 40-60 min prior to benserazide and L-DOPA challenge | NR | Improved: potentiated L-DOPA induced contralateral rotations; inhibited L-DOPA induced sensitization |
| 20655910; 2010 | Macaque | | | MPTP 2-3 mg/kg, SC, once weekly until stable Parkinsonism. Followed by induction of LID by L-DOPA after 8 weeks. | | | Preladenant 1 - 3 mg/kg; PO | | After MPTP, simultaneous with L-DOPA | NR | Improved: improved Parkinsonian scores, without dyskinesia |
| 20655910; 2010 | Capuchin monkey | | | Chronic haloperidol 0.3-3.0 mg/kg; PO | | | Preladenant 0.3-3.0 mg/kg; PO | | Given simultaneous with acute haloperidol challenge | NR | Improved: delayed onset of extrapyramidal syndrome |
| **10. Glial-derived neurotrophic factor: AAV2-Neurturin** | | | | | | | | | | | |
|  |  | | | **Model/Patient details** | | | | | | **Outcome** | |
| **PMID** | **Species** | | | **Model/Patient** | | | **Intervention** | | **Timing of intervention** | **Non clinical** | **Clinical** |
| 20970382; 2010 | Human | | | PD | | | AAV2-neurturin (5·4 × 10¹¹ vector genomes) injected bilaterally into the putamen | | Severe Parkinson’s disease | NR | No effect  I^o^ OM: UPDRS motor score at 12 months |
| 26061140; 2015 | Human | | | PD | | | Bilateral AAV2-neurturin injected; substantia nigra (2.0 × 10 ^11^ vector genomes) and putamen (1.0 × 10 ^12^ vector genomes) | | Severe Parkinson’s disease | NR | No effect  I^o^ OM: UPDRS motor score at 15-24 months |
| 17192932; 2006 | Macaque | | | MPTP: 3mg unilateral intracarotid injection 1X | | | AAV2-neurturin injected into the striatum and substantia nigra | | 4 days after MPTP | Preserved nigral neurons and striatal dopaminergic innervation, activated phospho-extracellular signal-regulated kinase | Improved: reduced MPTP-induced motor impairments; at 4 and 10 months after treatment |
| 23631873; 2013 | Rat | | | 6-OHDA; 20 μg unilateral striatal injection | | | AAV2-NRTN (CERE-120) to continuously express NRTN to either striatum or nigra alone or combined striatal/nigral exposure | | Prior to 6-OHDA by 2 to 6 weeks, or simultaneous with 6-OHDA | Neuroprotection of nigrostriatal neurons | NR |
| 17532642; 2007 | Rat | | | 6-OHDA; 20 μg unilateral striatal injection | | | CERE-120 delivery to the striatum | | Prior to 6-OHDA by 2 weeks | Neuroprotection of nigrostriatal neurons | Improved: behavioral recovery for at least 6 months |
| 7749731; 1995 | Rat | | | 6-OHDA; 8 μg unilateral substantia nigra injection | | | intranigral GDNF | | Prior to 6-OHDA by 24 hours | Neuroprotection of nigral neurons | NR |
| 8929429; 1996 | Rat | | | 6-OHDA; 20 μg unilateral substantia nigra injection | | | GDNF injected close to the substantia nigra | | 5 days after 6-OHDA | Long-term (4 months) protection of lesioned nigral neurons | No effect: no functional recovery in spontaneous motor behaviors |
| 9184114; 1997 | Rat | | | 6-OHDA; 9 μg bilateral intracranial, medial forebrain bundle, 1X | | | 250 microg GDNF or vehicle injected into the right lateral cerebral ventricle | | Two weeks after 6-OHDA | Neuroprotection of nigral neurons; increase in dopamine content within the substantia nigra and ventral tegmenta | Improved: locomotor ability |
| 9753113; 1998 | Rat | | | 6-OHDA; unilateral intracranial, medial forebrain bundle, 1X | | | GDNF was administered ipsilaterally above the substantia nigra and into the lateral ventricle | | Immediately prior to 6-OHDA | Neuroprotection of nigral neurons; increase in striatal dopamine content | Improved: prevented the development of amphetamine-induced rotations |

* Methodologic details of 70 interventional human and animal publications for PD – one publication is defined as a unique PMID. Several animal model publications utilized more than one species or contained more than one study.

**Non-clinical outcomes in animal models***:* Nigrostriatal morphology and immunohistochemistry, striatal biochemistry, biomarkers of oxidative damage and inflammation, DA-transporter autoradiography; striatal levels of creatine, phosphocreatine, ATP and lactate, striatal alpha synuclein immunohistochemistry, brain and plasma CoQ_10_ and Vit E, mitochondrial CoQ_10,_ mitochondrial activity, caspase -3, markers of inflammation, Striatal PPARg, UCP-2 and microNEET, plasma/CSF pioglitazone, MKK4 and JNK signaling, kinase-induced phosphorylation in T and B-lymphocytes, Binding Autoradiography, striatal pERK and deltafosB , SN electrophysiology

**Clinical outcomes in animal models***:* Actimetry, Abnormal involuntary movement scale, beam walk test, cylinder test, dyskinesia scale in NHPs, forced swim test, grip test, Morris water maze, open field test, Parkinsonian Disability scale in NHPs, paw test, rotarod test, two-way avoidance task.
