# Supplementary material for: Established patterns of animal study design undermine translation of disease-modifying therapies for Parkinson’s disease
Source: PLoS One. 2017 Feb 9;12(2):e0171790. doi: 10.1371/journal.pone.0171790 (PMC5300282; doi:10.1371/journal.pone.0171790)
Supplement: S4 Table — (DOCX) [file pone.0171790.s007.docx]

**S4 Table: Interventional studies in human and non-human animals**

|  | Search terms (PubMed) | Time period* | Interventional studies ****** | Symptomatic  ******* | Disease -modifying******* |
| --- | --- | --- | --- | --- | --- |
| **Animal studies** | | | |  |  |
| Mouse | “Mouse” and “Parkinson’s disease” | 2015 | 144 | 23 | 121 |
| Rat | “Rat” and “Parkinson’s disease” | 2015 | 93 | 27 | 66 |
| Marmoset | “Marmoset” and “Parkinson’s disease” | 2006-2015 | 36 | 30 | 6 |
| Macaque | “Macaque” and “Parkinson’s disease” | 2006-2015 | 56 | 31 | 25 |
| Vervet monkey | “Vervet ****” and “Parkinson’s disease” | 1986-2015 | 22 | 5 | 17 |
| Human | “Parkinson’s disease”, limited by Clinical trials | 1989-2015 | 94 | 63 | 31 |
| Total studies (Animal and Human) | | | **445** | **179** | **266** |

* Our intent was to collect at least 90 of the most recent studies for each species category (mouse, rat, non-human primates and human). Because of the large number of studies published each year using rodents, mouse and rat studies from 2015 only were included. In contrast, to acquire sufficient studies in humans and non-human primates, the search period was extended backwards by full years from December 1, 2015 in these species until the required number of intervention studies was achieved. A total of 425 individual publications (unique PMID’s) were included – because some individual papers included studies using more than one species, the total number of studies (445) exceeds the total number of individual publications.

** Only interventional studies were used for outcomes analysis - interventions were defined in the title or abstract of the study.

***Interventions were classified as symptomatic (defined as temporary amelioration of Parkinsonian signs or complications of dopaminergic treatment without altering the course of the disease) or potentially disease-modifying (alteration of disease course). Those interventions that are already approved for PD, related interventions within a similar class, and interventions such as exercise that have been used in PD but so far have not been shown to appreciably alter disease course) were classified as symptomatic. The remainder were classified as potentially disease-modifying.

**** Because of the variety of terms used to denote this species, additional synonyms for Vervet monkey were used – these included: Chlorocebus aethiops, Chlorocebus pygerythrus, Chlorocebus sabaeus, Cercopithecus sabeus, Cercopithecus aethiops Savannah Monkey, Grivet Monkey, African Green Monkey,
