# Supplementary material for: Established patterns of animal study design undermine translation of disease-modifying therapies for Parkinson’s disease
Source: PLoS One. 2017 Feb 9;12(2):e0171790. doi: 10.1371/journal.pone.0171790 (PMC5300282; doi:10.1371/journal.pone.0171790)
Supplement: S5 Table — (DOCX) [file pone.0171790.s008.docx]

**S5 Table: Methodologic details in MPTP (mice, non-human primates) and 6-OHDA (rats) models by intervention type**

| MPTP studies in non-human primates (N= 80) | | | | | | | | | | | | |
| --- | --- | --- | --- | --- | --- | --- | --- | --- | --- | --- | --- | --- |
| Study type (n) | Age (years, n) | Sex | MPTP Protocol | Time (weeks or months) between MPTP and intervention initiation (range, n) | | | Study design and reporting | | | | | |
|  |  |  |  | Prior | During | After | Average group size +/-sd (n=% reporting animal numbers) | Randomization (%) | Blinding (%) | Statistical test reported | Diet and housing | |
| Marmoset (n=30) | | | | | | | | | | | | |
| Symptomatic (n=27) | 1.5-8 years  (n=14)  Adult  (n=3)  NR = 11 | M (n=2)  F (n=3)  M, F (n=17)  NR = 5 | 2 mg/kg sid SC for 3 days (n=2)  2 mg/kg sid SC for 5 days (n=21)  0.7-1.5 mg/kg sid SC for 9-10 days (n=2)  2.5 mg/kg SC once weekly for 2-4 weeks (n=2) | 1 week (n=2) | n=2 | 2-36 weeks (n=23) | 5.9+/-1.6 (n=100%) | 11% | 66% | 100% | 96% | |
| Disease-modifying (n=3) | 1-3 years  (n=2)  NR = 1 | M (n=1)  F (n=1)  M, F (n=1) | 2 mg/kg sid SC for 5 days (n=1)  1 mg/kg sid SC for 8 days (n=1)  2 mg/kg SC over 1.5 years (n=1) | 1 week (n=1) | n=0 | 16 -22 weeks (n=2) | 5.5+/-0.7(n=100%) | 66% | 66% | 100% | 100% | |
| Vervet monkey (n=22) | | | | | | | | | | | | |
| Symptomatic (n=5) | Adult (n=4)  NR=1 | M (n=3)  F (n=2) | 0.3-0.6 mg/kg sid IM for 5 days (n=5) | n=0 | n=0 | 2-11 weeks (n=3)  NR =2 | 1.8+/-0.8(n=100%) | 0% | 0% | 90% | 60% | |
| Disease-modifying (n=17) | Adult (n=14)  NR= 3 | M (n=14)  F (n=1)(  NR=2 | 0.3-0.6 mg/kg sid IM for 5 days (n=16)  0.6 mg/kg IV unilateral carotid (n=1) | 1.5 weeks (n=1) | n=0 | 3-20 weeks (n=16) | 4.9+/-2.4(n=100%) | 11% | 47% | 64% | 52% | |
| Rhesus and cynomolgus macaques (n=28) | | | | | | | | | | | | |
| Symptomatic (n=17) | 4-8.2 years (n=14)  Adult (n=3)  NR = 1 | M (n=5)  F (n=11)  M, F (n=1) | 0.2-0.6 mg/kg IM at intervals of 1 to 7 or more days to effect (n=3)  0.2-0.8 mg/kg IV at intervals of 1 or more days to effect (n=9)  0.5 mg/24 hrs SC minipump to effect (n=4)  0.2 mg/kg SC daily for 8-30 days (n=1) | n=0 | n=2 | 2.5-6.6 months (n=15)  NR =2 | 5.6+/-2.1 (n=100) | 5% | 64% | 100% | | 100% |
| Disease-modifying (n=11) | 5-12 years (n=8)  Adult (n=1)  NR = 2 | M (n=6)  F (n=4)  M, F (n=1) | 0.2-1.5 mg/kg IC unilaterally once (n=9)  0.2-0.8 mg/kg IV at intervals of 1 or more days to effect (n=1)  NR=1 | n=0 | n=1 | 1.5-6 months (n=10) | 4.3+/-1.6 (n=100%) | 80% | 80% | 100% | | 72% |
| MPTP studies in mice (N=92) | | | | | | | | | | | | |
| Strain (n) | Age in average weeks +/-sd ( n) | Sex | MPTP Protocol | Time (days) between MPTP and intervention initiation(mean +/-sd) | | | Study design and reporting | | | | | |
|  |  |  |  | Prior | During | After | Average group size +/-sd (n=% reporting animal numbers) | Randomization (%) | Blinding (%) | Statistical test reported | | Diet and housing |
| Symptomatic (n=5) | | | | | | | | | | | | |
| C57BL and related (n=5) | Mean 8.6 +/-2.5  (n=4)  NR (n= 1) | M (n=5) | 4.75-40 mg/kg 2-4 X IP over 1 day (n=1)  20-30 mg/kg once daily IP for 4 days (n=1)  20-37.5 mg/kg once daily IP for 5 days (n=1)  20 mg/kg once daily IP for 28 days (n=1) | 2.7 (4.7)  n= 3 | n=0 | 1.4 (2.3)  n=2 | 8.4 +/3.1 (n=100%) | 20% | 0% | 100% | | 40% |
| Disease-modifying (n=87) | | | | | | | | | | | | |
| C57BL and related (n=81)  BALB/c (n=2)  Kunming (n=1)  NR=3 | Mean 10.1 +/-3  (n=50)  Adult (n=12)  NR (n=15) | M (n=74)  F (n=1)  NR (n=12) | 4.75-40 mg/kg 2-4 X IP over 1 day (n=33)  18-40 mg/kg 2-4X IP/ SC over 2 days (n=2)  20-30 mg/kg once daily IP for 4 days (n=4)  20-37.5 mg/kg once daily IP for 5 days (n=25)  20-30 mg/kg once daily IP/SQ for 7 days (n=10)  30 mg/kg once daily IP for 8 days (n=2)  20 mg/kg SQ 5 injections over 9 days (n=1)  10-40 mg/kg once daily IP/SQ for 14 days (n=3)  20 mg/kg tid IP for 21 days (n=1)  20 mg/kg once daily IP for 28 days (n=1)  25 mg/kg MPTP (plus probenecid, 250 mg/kg) SQ/IP twice weekly over 35 days (n=5) | 7.5 (11.1)  n= 55 | n=5 | 2.6 (4.2) N=27 | 8.0 +/3.1 (n=90%) | 34% | 12% | 98% | | 53% |
| 6-OHDA studies in rats (N=44) | | | | | | | | | | | | |
| Strain (n) | Age in average weeks +/-sd ( n) | Sex | 6-OHDA Protocol | Time (days) between 6-OHDA and intervention (mean +/-sd, n) | | | Study design | | | | | |
|  |  |  |  | Prior | During | After | Average group size +/-sd (n=% reporting animal numbers) | Randomization (%) | Blinding (%) | Statistical test reported | | Diet and housing |
| Symptomatic (n=15) | | | | | | | | | | | | |
| Sprague-Dawley (n=11)  Wistar (n=2)  Long-Evans (n=1)  Hannover (n=1) | 34.3 +/- 33.1 (n = 16) | M (n=13)  F (n=3) | 3-30 µg given once, unilateral, intracranial (n=16) | 15.8 +/-3.5  (n= 3) | N=0 | 73.6 +/- 154.9  (n= 13) | 9.8 +/3.6 (n=87%) | 27% | 6.1% | 100% | | 93% |
| Disease-modifying (n=29) | | | | | | | | | | | | |
| Sprague-Dawley (n=10)  Wistar (n=12)  Fischer 344 (n=1)  NR=7 | 10.6 +/- 2.2 (n = 4)  Adult (n=9)  NR = 17 | M (n=22)  F (n=6)  NR (n=1) | 3-30 µg given once, unilateral, intracranial (n=26)  7 µg given once daily, unilateral, intracranial over 3 days (n=1)  20 µg given twice one week apart, unilateral, intracranial (n=1)  NR=2 | 29.3 +/-57.1  (n= 10) | N=0 | 13.7 +/- 11.1  (n= 18) | 8 +/- 2.3 (n=100%) | 52% | 6.9% | 96% | | 93% |

N = number of published reports in analysis; n = number of studies reporting a given variable. The sum of n may exceed N if more than one experiment was done, or more than one species was used in a study; NR = not reported

Time between administration of the toxin and treatment with the intervention is given over three categories (Prior, during or after toxin administration). Animals given the intervention in more than time period are counted in each relevant category.

Sample size calculation: 0% across all species
